# Anthropomorphic tissue-mimicking phantoms for oximetry validation in multispectral optical imaging

**DOI:** 10.1117/1.JBO.30.7.076006

**Published:** 2025-07-17

**Authors:** Kris K. Dreher, Janek Gröhl, Friso Grace, Leonardo Ayala, Jan-Hinrich Nölke, Christoph J. Bender, Melissa J. Watt, Katie-Lou White, Ran Tao, Wibke Johnen, Minu D. Tizabi, Alexander Seitel, Lena Maier-Hein, Sarah E. Bohndiek

**Affiliations:** a German Cancer Research Center (DKFZ), Division of Intelligent Medical Systems (IMSY), Heidelberg, Germany; b Heidelberg University, Faculty of Physics and Astronomy, Heidelberg, Germany; c University of Cambridge, CRUK Cambridge Institute, Cambridge, United Kingdom; d University of Cambridge, Department of Physics, Cambridge, United Kingdom; e University of St Andrews, School of Physics and Astronomy, St Andrews, United Kingdom; f NCT Heidelberg, a partnership between DKFZ and University Hospital Heidelberg, National Center for Tumor Diseases (NCT), Heidelberg, Germany; g Heidelberg University, Faculty of Mathematics and Computer Science, Heidelberg, Germany; h DKFZ, Division of Medical Physics in Radiation Oncology, Heidelberg, Germany; i Heidelberg Institute for Radiation Oncology (HIRO), National Center for Radiation Research in Oncology (NCRO), Heidelberg, Germany; j Heidelberg University, Medical Faculty, Heidelberg, Germany

**Keywords:** anthropomorphic phantoms, optical imaging, oximetry, photoacoustic imaging, hyperspectral imaging

## Abstract

**Significance:**

Optical imaging of blood oxygenation (${\mathrm{sO}}_{2}$) can be achieved based on the differential absorption spectra of oxy- and deoxyhemoglobin. A key challenge in realizing clinical validation of the ${\mathrm{sO}}_{2}$ biomarkers is the absence of reliable ${\mathrm{sO}}_{2}$ reference standards, including test objects.

**Aim:**

To enable quantitative testing of multispectral imaging methods for assessment of ${\mathrm{sO}}_{2}$ by introducing anthropomorphic phantoms with appropriate tissue-mimicking optical properties.

**Approach:**

We used the stable copolymer-in-oil base material to create physical anthropomorphic structures and optimized dyes to mimic the optical absorption of blood across a wide spectral range. Using 3D-printed phantom molds generated from a magnetic resonance image of a human forearm, we molded the material into an anthropomorphic shape. Using both reflectance hyperspectral imaging (HSI) and photoacoustic tomography (PAT), we acquired images of the forearm phantoms and evaluated the performance of linear spectral unmixing (LSU).

**Results:**

Based on 10 fabricated forearm phantoms with vessel-like structures featuring five distinct ${\mathrm{sO}}_{2}$ levels (between 0 and 100%), we showed that the measured absorption spectra of the material correlated well with HSI and PAT data with a Pearson correlation coefficient consistently above 0.8. Further, the application of LSU enabled a quantification of the mean absolute error in ${\mathrm{sO}}_{2}$ assessment with HSI and PAT.

**Conclusions:**

Our anthropomorphic tissue-mimicking phantoms hold potential to provide a robust tool for developing, standardising, and validating optical imaging of ${\mathrm{sO}}_{2}$.

## 1 Introduction

Oxyhemoglobin (${\mathrm{HbO}}_{2}$) and deoxyhemoglobin (Hb) are critical endogenous contrast agents that enable noninvasive measurement of blood oxygen saturation (${\mathrm{sO}}_{2}$), also referred to as oximetry, due to their distinct optical absorption spectra. Oximetry methods are invaluable for a range of clinical applications throughout the patient care pathway, from diagnosis to treatment planning and monitoring of treatment response.^1^^–^^5^ Pulse oximetry is the most widely available of these tools, which employs red and infrared light to noninvasively estimate ${\mathrm{sO}}_{2}$ for bulk tissue at a single measurement site, such as a fingertip.^1^

To obtain spatially resolved information, for example, in image-guided surgery, imaging modalities such as hyperspectral imaging (HSI) and photoacoustic tomography (PAT) are used. HSI and PAT typically involve making wavelength-resolved measurements across the visible and near-infrared spectral range.^1^ HSI uses light diffusely reflected from the tissue to map ${\mathrm{HbO}}_{2}$ and Hb signals near the tissue surface,^6^^,^^7^ whereas PAT combines pulsed laser illumination and ultrasonic detection to probe deeper tissue layers.^8^^,^^9^ In both techniques, deriving ${\mathrm{sO}}_{2}$ from spectral data commonly relies on linear spectral unmixing (LSU),^10^^,^^11^ assuming that the optical absorption responsible for the image contrast is a linear combination of the absorption spectra of all contrast agents present at any given point weighted by their concentration.

Accurately quantifying ${\mathrm{sO}}_{2}$ in HSI and PAT is challenging because a range of assumptions are made in both the datacube (x,y,λ) reconstruction and spectral analysis pipeline that can lead to corruption of the measured tissue biomarkers. For example, in HSI, signals can be distorted due to additional optical interactions, such as fluorescence, whereas in PAT, depth-dependent signal attenuation arises, known as spectral coloring. In both modalities, patient motion and skin tone bias can introduce further complexity.^10^^,^^12^ Consequently, developing robust oximetry calibration methods for HSI and PAT remains an active area of research.^13^^–^^16^

For the development and rigorous validation of any scientific method, including oximetry, a reliable performance measure or reference is essential. In oximetry, however, the principal challenge is that an *in vivo* ground truth for ${\mathrm{sO}}_{2}$ is not available noninvasively with current technology. Therefore, many studies only rely on qualitative visualizations or measurement of relative changes in the same individual or specimen over time, rather than calibrating for absolute ${\mathrm{sO}}_{2}$.^17^^–^^19^ Validation approaches can take several forms. Validation can be developed by comparison to simulated data; however, these often fail to generalize when applied to *in vivo* tissue.^20^ Adding a level of complexity, experimental data obtained from bulk test objects (“phantoms”) made with a mixture of blood, hemoglobin, or other biological solutions^21^^,^^22^ can closely mimic tissue spectra and facilitate validation, but typically do not allow well-controlled adjustment of ${\mathrm{sO}}_{2}$, lack internal structure, tend to be unstable over time, and are prone to bacterial contamination.^23^^,^^24^ Addressing some of these limitations, blood flow phantoms allow for chemical manipulation of ${\mathrm{sO}}_{2}$ as blood flows through a circuit while being imaged.^25^^,^^26^ Nevertheless, blood flow circuits rely on ancillary reference measurements (e.g., partial pressure probes or offline oximeters) to provide a gold standard reference. They are typically simple in their structure, e.g., a tube flowing through a slab of base material and thus lack anatomical variability, which is not only beneficial to train deep learning methods but also allows for a detailed analysis of oximetry methods in depth and their performance dependent on different amounts and positions of absorbing structures.

Structurally stable, anthropomorphic phantoms with tissue-mimicking optical properties over a broad wavelength range remain rare.^27^ Obtaining oximetry estimations from anthropomorphic phantoms typically requires embedding a flow circuit within a complex material composition, which is prohibitively challenging. To overcome this limitation and tackle the challenge of oximetry validation in HSI and PAT, we introduce an anthropomorphic phantom design that mimics the morphology of a human forearm and includes absorbing dyes that replicate the absorption coefficient ($\mu_{a}$) characteristics of ${\mathrm{HbO}}_{2}$ and Hb between 700 nm and 850 nm. We first selected and optimized dye proxies for Hb and ${\mathrm{HbO}}_{2}$. We then created 3D-printable molds derived from human forearm imaging to achieve morphologically relevant geometry, which are available open-source. Using a copolymer-in-oil base material, we created structurally stable anthropomorphic forearm phantoms with optical characteristics confirmed via simulation studies and signal correlation analyses. Finally, we demonstrated the value of the created phantoms for oximetry validation in HSI and PAT. These phantoms provide a versatile, morphologically realistic test platform for oximetry validation in the near-infrared. We see another major contribution of this work that in addition to the specific structures and absorptions we targeted, this paper presents a general workflow from external data of human anatomy and a specific target tissue, in our case, magnetic resonance imaging (MRI) data and blood vessels, respectively, to structurally stable tissue-mimicking phantoms.

## 2 Materials and Methods

**Fig. 1:**
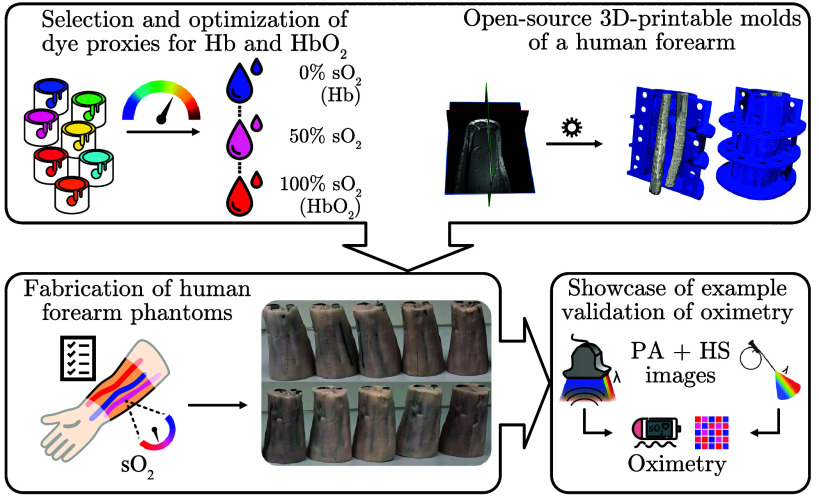
Workflow and key contributions of this study. (Top) First, 26 dyes were investigated to mimic the absorption spectra of oxyhemoglobin (${\mathrm{HbO}}_{2}$) and deoxyhemoglobin (Hb) in the wavelength (λ) range of 700 to 850 nm. After a selection and optimization process, two proxy dyes that could be mixed to five levels of oxygen saturation (${\mathrm{sO}}_{2}$) (0%, 30%, 50%, 70%, and 100%) were found. Second, for realistic tissue morphology, a 3D-printable mold was created based on an open-source magnetic resonance (MR) image of a human forearm. (bottom) Third, ten forearm phantoms were fabricated and finally, photoacoustic (PA) and hyperspectral (HS) images were acquired and we show that example images of these can be used to validate oximetry methods.

Combining dye proxies with anthropomorphic phantom designs in this study enables a new approach to oximetry validation (Fig. 1).

### 2.1 Forearm Phantom Fabrication

#### 2.1.1 Phantom material

The base phantom material was prepared according to protocols outlined by Hacker et al.^28^ and Gröhl et al.^29^ Briefly, for each ∼80  mL batch of base material, 76.5 mg of titanium dioxide (${\mathrm{TiO}}_{2}$, Sigma Aldrich 232033-100 g) was sonicated in a water bath together with 50 mL of mineral oil (Sigma Aldrich 330779-1L) until completely dispersed. Next, 12.57 g of polystyrene-block-poly(ethylene-ran-butylene)-block-polystyrene (SEBS, Sigma Aldrich 200557-250G) and 1 g of butylated hydroxytoluene (HT, Sigma Aldrich W218405-1KG-K) were added to the oil. The mixture was heated in a silicone oil bath at 160°C for about 45 min, stirred every 10 min, and allowed to liquefy fully. Finally, the beaker was placed in a vacuum chamber to remove any residual air bubbles. A more detailed manufacturing protocol is provided in the Supplementary Notes S1 in the Supplementary Material.

#### 2.1.2 Optical property characterization

The optical properties of the phantom materials were quantified using a double-integrating sphere (DIS) system (according to the method of Pickering et al.^30^ with the system described in Hacker et al.^28^). The DIS system measured total reflectance and transmittance over a wavelength range of 700 to 850 nm, and the resulting data were processed with the inverse adding-doubling (IAD)^31^ algorithm to obtain $\mu_{a}$ and scattering coefficient ($\mu_{s}$).^32^ The refractive index was set to n=1.4, and the anisotropy factor to g=0.7, as suggested by Jones and Munro.^33^ For each material batch, two optical sample slabs were fabricated. The thickness of each slab was measured five times at three different locations (top, middle, and bottom) using a digital calliper and provided as input to the IAD algorithm. Subsequently, each location was measured from both the front and back with the DIS system, producing 12 individual data points per wavelength.

#### 2.1.3 Proxy dyes for oxy- and deoxyhemoglobin

After starting with dyes that were already used in combination with gel-wax phantoms,^34^ a total of 26 candidate dyes were evaluated for their optical properties in the abovementioned base material between 700 and 850 nm (Figs. S1–S3 in the Supplementary Material, Table S1 in the Supplementary Material). The $\mu_{a}$ spectra measured with the DIS system served as inputs to a non-negative least square (NNLS) optimization, which aimed to identify a linear combination of dyes whose summed absorption profiles closely match those of ${\mathrm{HbO}}_{2}$ and Hb. As target spectra, we used the data available at Ref. 35. Based on these results, the most promising dyes were selected and further refined by iteratively adjusting concentrations and re-measuring $\mu_{a}$.

#### 2.1.4 3D forearm model

**Fig. 2:**
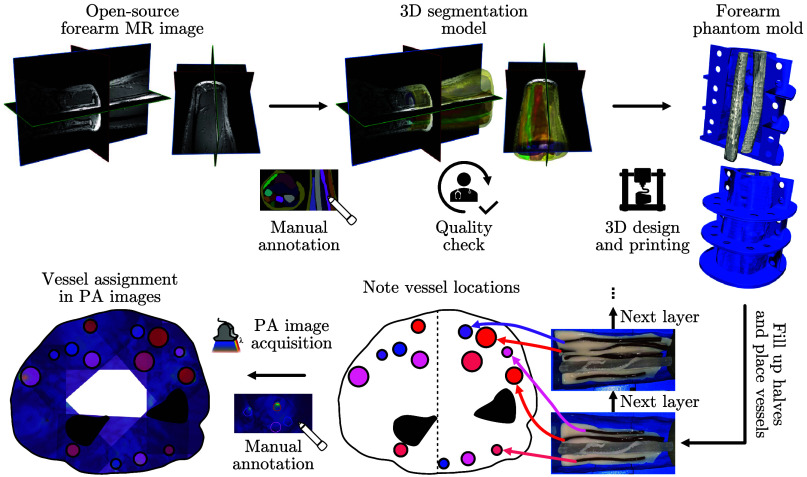
3D-printable models were constructed from an open-source magnetic resonance (MR) image for a structured phantom fabrication and annotation process. From top left to top right: The MR image was manually segmented for bones, muscles, and fat. A physician performed a quality check ensuring morphological correctness. Based on this segmentation, a 3D-printable model of the outer hull of the forearm has been designed and printed including the two bones, radius and ulna, that will stay in the mold as positives. From bottom right to bottom left: Each half of the mold is filled successively with background material and vessels are placed, which are annotated in a cross-sectional sketch of the mold. Finally, after acquiring photoacoustic (PA) images from multiple angles, each vessel in the PA images can be assigned to its corresponding oxygen saturation during manual annotation.

A high-resolution MRI dataset of the human forearm was obtained from Kerkhof et al.^36^ From these data, muscle and bone structures were segmented, and the segmentation was verified by a physician (MDT). The outer hull and bones were then 3D-printed to serve as mold and embedded features, respectively (Fig. 2). The digital model for the mold was designed and constructed using Autodesk Inventor 2024 and Geomagic Freeform. An Objet 500 Connex (Stratasys, Ltd., Eden Prairie, Minnesota, USA) was used for 3D printing. Specifically, VeroCyan™ was used to fabricate the external mold, whereas VeroClear™ was chosen to print the bones, which remained inside each phantom as positive structures. Visual inspections and photoacoustic measurements of VeroClear™ indicate negligible $\mu_{a}$ and $\mu_{s}$; thus, we did not opt for a challenging and expensive full optical characterization.

#### 2.1.5 Phantom fabrication process

Ten forearm phantoms were fabricated, each containing background material and 14 embedded vessels. The vessels were created by drawing phantom material into tubes with diameters of 3, 4, 5, or 6 mm, producing 140 vessels in total (28 per ${\mathrm{sO}}_{2}$ level, 7 per diameter). The minimum vessel diameter we could fabricate was 3 mm, which coincides with the upper range of the radial artery, the largest superficial vessel typically visible in the forearm. Therefore, our vessel diameters are larger than anatomically expected. At least one vessel for each ${\mathrm{sO}}_{2}$ level was positioned near the surface (“superficial vessel”) in each phantom. Dye mixtures within the vessels were determined based on the outcome of the NNLS optimization. Particular attention was paid to achieving an isosbestic point near 800 nm. For mixing intermediate ${\mathrm{sO}}_{2}$ levels, the base dyes were accurately weighed, and the resulting ratios were verified by measuring $\mu_{a}$ spectra and applying LSU. This two-step approach ensured reliable verification of the intended mixing ratios.

Nine of the phantoms were manufactured with background materials spanning three ${\mathrm{sO}}_{2}$ levels (0%, 50%, and 100%) and their volumes as fractions of the respective material batch (1%, 2.5%, and 4%), yielding 3×3 combinations. An additional out-of-distribution (OOD) phantom was created with three distinct combinations of volume fraction and ${\mathrm{sO}}_{2}$ (0.5%, 100%), (5%, 0%), and (3%, 70%) (Table S2 in the Supplementary Material). Optical properties were verified for each background material (Figs. S4–S7 in the Supplementary Material). The fabrication proceeded by splitting the 3D-printed mold into two halves and pouring two 100-mL batches of the phantom mixture in layers. Vessels were placed incrementally within each half after each layer, and their approximate positions were documented. To ensure that the overall coverage of vessel locations matches with human forearms, we compared them by plotting the vessel locations in both humans from an in-house dataset^37^ and phantoms in Fig. S8 in the Supplementary Material. Once both halves were filled and all 14 vessels were in place, the two parts of the mold were joined, and any remaining cavity in the center was filled with the residual phantom material.

### 2.2 Optical Imaging Techniques

#### 2.2.1 Photoacoustic tomography

All phantoms were scanned using the MSOT Acuity Echo system (iThera Medical GmbH, Munich, Germany) in a water bath using the wavelengths from 700 to 850 nm in steps of 10 nm. Each of the ten phantoms was imaged at three predefined locations, and for each location, eight angular views were acquired in 45-deg increments around the phantom. This arrangement resulted in 24 images per phantom, for a total of 240 images overall. The phantoms were mounted on a rotational stage within the water bath to facilitate consistent data acquisition across all angles and locations.

The acquired time-series data were corrected for laser energy (Fig. S9 in the Supplementary Material) and filtered using a bandpass with cutoffs at 50 kHz and 20 MHz. The 700-nm wavelength was excluded from subsequent analysis due to laser instability. Reconstructions were performed with a delay-and-sum algorithm implemented in the open-source toolkit for simulation and image processing for photonics and acoustics (SIMPA),^38^ specifying a speed of sound of $1497.4\;{\mathrm{ms}}^{-1}$ and a voxel resolution of 0.1 mm. After reconstruction, a Hilbert transform was applied for envelope detection. The speed of sound was chosen to enable coregistration with concurrently acquired ultrasound images. The photoacoustic (PA) images shown in this work are the unnormalized results of this reconstruction algorithm.

#### 2.2.2 Hyperspectral imaging

All phantoms were imaged using the Tivita 2.0 camera (Diaspective Vision GmbH, Am Salzhaff, Germany). The wavelengths from 500 to 1000 nm in steps of 5 nm were imaged, but for consistency with PAT, we used the wavelengths from 700 to 850 nm in steps of 10 nm for analysis in this work. Each phantom was placed on a rotational stage and scanned at eight angular positions in 45-deg increments. Before each capture, the camera was refocused to ensure clear images. This procedure yielded eight hyperspectral (HS) images per phantom, for a total of 80 images across all phantoms. The acquired HS images were automatically corrected for white and dark references using the open-source htc^39^ software.

### 2.3 Quality Assurance

To ensure that our phantoms faithfully replicate the intended optical and acoustic properties, we performed two key evaluations: simulation studies to investigate the effects of air bubbles and speed of sound variations on PA images and signal correlation analyses to confirm the relationship between measured signals and the known absorption spectra at varying ${\mathrm{sO}}_{2}$ levels.

#### 2.3.1 Simulation studies

Ultrasound segmentations including observed air bubbles were used to simulate phantom images, comparing vessel spectra with and without air inclusions. In addition, a sensitivity analysis on speed of sound variations (±50 and $\pm 100\;{\mathrm{ms}}^{-1}$ around the assumed speed of sound $1470\;{\mathrm{ms}}^{-1}$) was performed. Because the main focus of this work is on the optical properties, refer to Sec. S3 in the Supplementary Material for detailed experiment descriptions and results in Figs. S16–S25. Briefly, all simulations were conducted with SIMPA,^38^ employing MCX^40^ for photon transport and k-wave^41^ for acoustic wave propagation. Each simulation used a digital device twin of the MSOT Acuity Echo, and a digital tissue twin constructed from manual segmentations of five representative phantom PA images. The vessels and background regions in these digital twins were assigned the absorption and scattering coefficients obtained via the DIS system.

#### 2.3.2 Signal correlation

PA signals (S) are proportional to the product of the Grüneisen parameter (Γ), $\mu_{a}$, and the local fluence (Φ): $S\propto \Gamma \mu_{a}\Phi$.^8^ Because the Grüneisen parameter of the mixed dyes could be different such that the PAT signal does not correlate linearly with the $\mu_{a}$,^42^ we investigated whether the measured $\mu_{a}$ for different ${\mathrm{sO}}_{2}$ levels correlates linearly with the PA signal. The processing steps included

• Vessel segmentation: Superficial vessels were segmented as regions of interest (ROIs), and the top 5% of brightest pixels in each vessel region were averaged.
• Spectrum fitting: A linear regression (one multiplicative factor plus one offset) was applied to match the expected absorption spectrum from 710 to 850 nm.
• Correlation analysis: The Pearson correlation coefficient (R value) between the measured PA spectrum and the known absorption spectrum was determined for each vessel.

The HS signal (I) is formed from the diffusely reflected fraction of light that is neither absorbed nor scattered out of the detection path. A common method to approximate $\mu_{a}$ from reflectance data uses the Lambert-Beer law, where $\mu_{a}\propto -\log(I)$.^43^ We therefore checked whether the measured $\mu_{a}$ for different ${\mathrm{sO}}_{2}$ levels correlated with the HS signal. The processing steps include

• Region selection: ROIs were chosen within superficial vessels, avoiding specular highlights and vessel edges to minimize cross-talk from the surrounding tissue.
• Lambert-Beer approximation: An approximate absorption spectrum was obtained by applying ${\hat{\mu }}_{a}=-\log(I)$.
• Spectrum fitting: A linear regression (one multiplicative factor plus one offset) was applied to match the expected absorption spectrum from 700 to 850 nm.
• Correlation analysis: The R value was computed to assess the agreement between the derived absorption spectrum and the known phantom absorption.

### 2.4 Oximetry Method Validation

To quantify the accuracy of our phantom-based oximetry measurements, the derived spectra (both with and without signal correlation) were used as inputs to an LSU algorithm.^10^^,^^11^ The performance of LSU was assessed by calculating the mean absolute error (MAE) in estimated ${\mathrm{sO}}_{2}$ levels. In the PAT experiments, LSU performance was also evaluated as a function of depth. In addition, for PAT data, a fluence compensation step was introduced before LSU in simulation studies, where the reconstructed image was divided by the estimated fluence to account for optical attenuation effects in the PA signal. Finally, errors were computed at the most granular level—individual tissue-type instances (e.g., each vessel)—to obtain the mean, confidence interval, and standard deviation. These metrics were then successively aggregated following the hierarchical data structure: across vessels, across tissue types, and ultimately across all phantoms.

## 3 Results

### 3.1 Two Dyes Were Found to Mimic the Optical Absorption of Hemoglobin Across the Full Range of Oxygenations

**Fig. 3:**
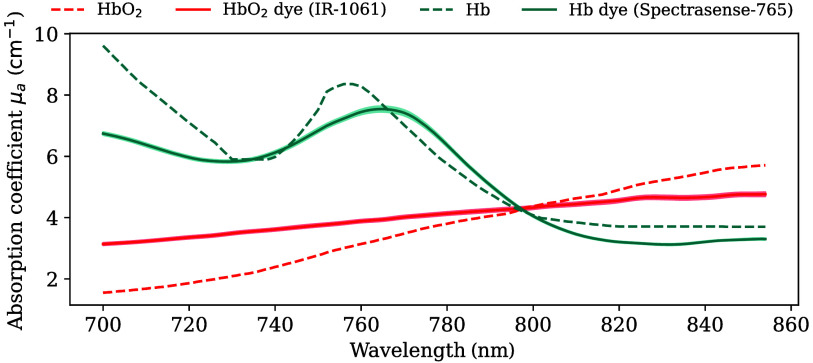
IR-1061 and Spectrasense-765 can mimic the absorption characteristics of the blood. Dashed lines indicate the absorption coefficient ($\mu_{a}$) spectra of oxyhemoglobin (${\mathrm{HbO}}_{2}$) and deoxyhemoglobin (Hb). Solid lines indicate the measured absorption spectra of the proxy dyes. Bands around the measured spectra indicate the standard deviation across the 12 measurement points for each optical sample slab. Mean absolute errors between targets and proxy dyes are 0.78 and $0.64\;{\mathrm{cm}}^{-1}$ for ${\mathrm{HbO}}_{2}$ and Hb, respectively.

Following the dye testing and optimization process, IR-1061 and Spectrasense-765 were identified as the closest proxies for blood absorption between 700 and 850 nm (Fig. 3, derived $\mu_{s}$ in Fig. S10 in the Supplementary Material) with Spectrasense-765 closely reproducing key features of Hb (local minimum at 730 nm, local maximum at 760 nm). Although IR-1061 exhibited a less pronounced slope than ${\mathrm{HbO}}_{2}$, the measured absorption still increased monotonically with wavelength. Notably, the isosbestic point for the two dyes was located at ∼800  nm, mirroring that of ${\mathrm{HbO}}_{2}$ and Hb. The R values to their respective target spectra can be found in Table S3 in the Supplementary Material.

### 3.2 Five Oxygen Saturation Levels with Characteristics Similar to Blood Were Derived

**Fig. 4:**
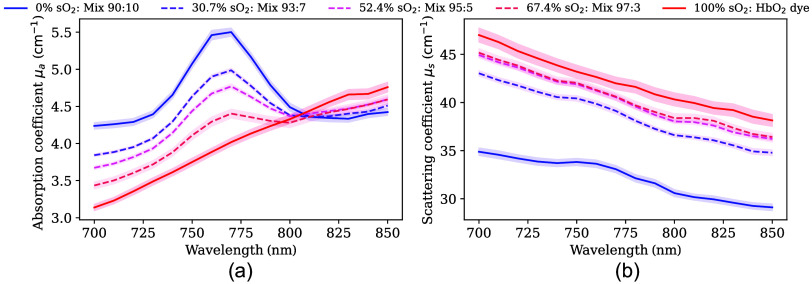
Five oxygen saturation (${\mathrm{sO}}_{2}$) levels were used for forearm phantom fabrication. Based on IR-1061 and Spectrasense-765, five ${\mathrm{sO}}_{2}$ levels (in %) were derived with the respective mixture ratios of 100:0, 97:3, 95:5, 93:7, and 90:10. Panels (a) and (b) represent the absorption coefficient ($\mu_{a}$) and scattering coefficient ($\mu_{s}$), respectively. Solid lines are the spectra that are used as endmembers (0% ${\mathrm{sO}}_{2}$ and 100% ${\mathrm{sO}}_{2}$) for linear spectral unmixing (LSU). Dashed lines represent the intermediate levels, and the corresponding percentages in the legend are the LSU results when using the solid lines as endmembers. Bands around the spectra indicate the standard deviation across the 12 measurement points for each optical sample slab.

By varying the mixing ratios of IR-1061 and Spectrasense-765, five levels of ${\mathrm{sO}}_{2}$ were created that approximate blood-like characteristics (Fig. 4; DIS reflectance and transmittance in Fig. S11 in the Supplementary Material). Although the relationship between dye concentrations and resulting $\mu_{a}$ spectra is highly nonlinear, a relatively linear trend was observed when transitioning from a 90:10 to a 100:0 dye ratio (representing 0% to 100% ${\mathrm{sO}}_{2}$ levels). Intermediate mixing ratios (e.g., 93:7, 95:5, and 97:3) then produced five distinct ${\mathrm{sO}}_{2}$ values: 0%, 30%, 50%, 70%, and 100%. Taking 90:10 and 100:0 as LSU endmembers yielded intermediate values of 30.7%, 52.4%, and 67.4% ${\mathrm{sO}}_{2}$.

### 3.3 Signal Correlation Shows Good Agreement with Measured Absorption Spectra

**Fig. 5:**
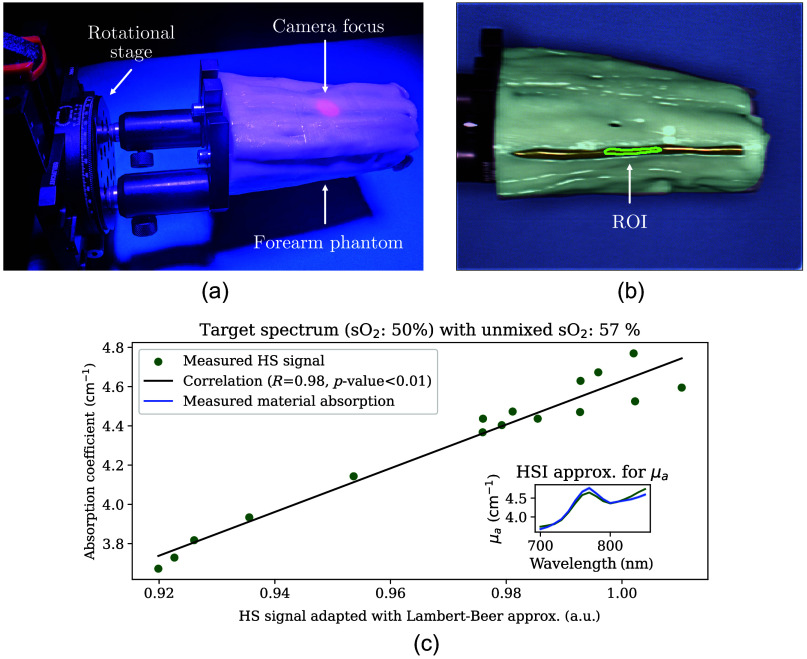
Measurement setup for hyperspectral imaging (HSI) (a), corresponding image (b), and signal correlation of the 50% oxygen saturation (${\mathrm{sO}}_{2}$) superficial vessel (c). The phantoms were mounted on a rotational stage, and the camera was adjusted for each phantom such that the middle of the phantoms was in focus (red dot). Images from eight angles in steps of 45 deg were acquired per phantom. Panel (b) shows an example of an RGB-reconstructed image using the spectral range of 530 to 725 nm including a region of interest (ROI) indicated by yellow margins in the middle of a superficial vessel. The black solid lines in panel (c) represent the resulting linear regression function with the corresponding Pearson correlation coefficient (R value). The inset plot shows the measured absorption (blue) and estimated absorption (green, using the correlation function) as qualitative confirmation.

The HSI setup [Fig. 5(a)] captures a top-down view of each phantom [Fig. 5(b)]. Analysis of the signal in the denoted ROI for the representative case with 50% ${\mathrm{sO}}_{2}$ demonstrated an extremely close match with the expected spectra [Fig. 5(c)].

**Fig. 6:**
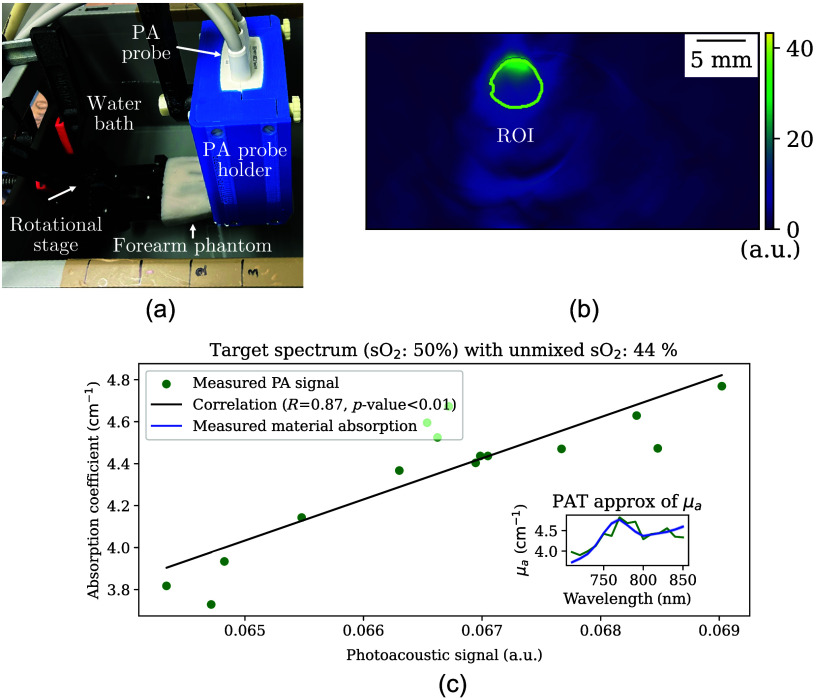
Measurement setup for photoacoustic tomography (PAT) (a), corresponding image (b), and signal correlation of the 50% oxygen saturation (${\mathrm{sO}}_{2}$) superficial vessel (c). The phantoms were mounted on a rotational stage in a water bath, and images were acquired from eight angles in steps of 45 deg in three distinct locations along the phantoms. For each measurement location, the PAT probe, which was attached to a mechanical arm to minimize motion during image acquisitions, was adjusted such that it was approximately in the middle of the phantoms and 2 mm above the uppermost point of the phantom. Panel (b) shows an example PA image at 800 nm including a region of interest (ROI) indicated by yellow margins in the middle of a superficial vessel. The black solid lines in panel (c) represent the resulting linear regression function with the corresponding Pearson correlation coefficient (R value). The inset plot shows the measured absorption (blue) and estimated absorption (green, using the correlation function) as qualitative confirmation.

The PAT system [Fig. 6(a)] provides cross-sectional images of the phantoms [Fig. 6(b)]. The PAT correlation analysis in Fig. 6(c) also shows high concordance with the expected absorption, although a higher degree of noise is seen in the PAT data compared with HSI. Analogous results were obtained from other imaged forearm phantoms across the range of defined ${\mathrm{sO}}_{2}$ levels, including images with more than one embedded vessel in the cross-sectional image (Figs. S12–S15 in the Supplementary Material). The correlation coefficient R value between the imaging signals and the measured absorption spectra in superficial vessels across the five example phantoms all exceeded 0.8, indicating strong agreement (Table 1).

**Table 1:** Pearson correlation coefficients (R value) for linear regression of hyperspectral imaging (HSI) and photoacoustic tomography (PAT) signal and absorption correlation.

	0% ${\mathrm{sO}}_{2}$	30% ${\mathrm{sO}}_{2}$	50% ${\mathrm{sO}}_{2}$	70% ${\mathrm{sO}}_{2}$	100% ${\mathrm{sO}}_{2}$
HSI	0.85	0.99	0.98	0.90	0.97
PAT	0.85	0.83	0.87	0.93	0.99

### 3.4 Dyes Do Not Exhibit Any Significant Long-Term Optical Degradation

To indirectly examine the long-term optical stability of the used dyes, we re-recorded the HS images described above in Sec. 3.3 more than 1 year later and performed the same correlation analysis between the original and the re-recorded HS signals. The R values in Table 2 all exceed 0.95 and thus show very strong agreement. Plots with all measured spectra and their correlations are provided in the Figs. S26–S30 in the Supplementary Material.

**Table 2:** Pearson correlation coefficients (R values) for linear regression of the original and re-recorded hyperspectral (HS) signal (measured over 1 year later) correlations.

	0% ${\mathrm{sO}}_{2}$	30% ${\mathrm{sO}}_{2}$	50% ${\mathrm{sO}}_{2}$	70% ${\mathrm{sO}}_{2}$	100% ${\mathrm{sO}}_{2}$
R-value	0.98	0.97	1.00	0.96	0.99

### 3.5 Forearm Phantoms Can Be Used for Validation of Oximetry Methods

**Fig. 7:**
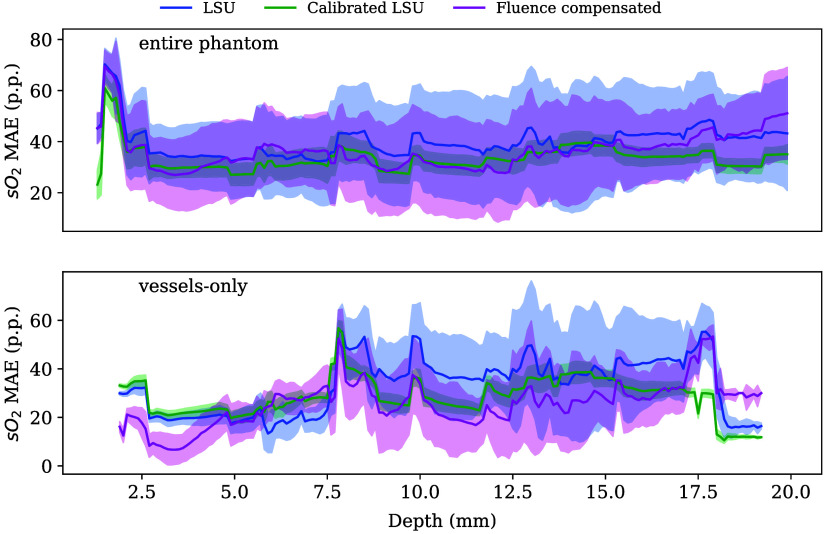
Error analysis of three oximetry methods for photoacoustic tomography as a function of depth. Mean absolute error (MAE) in percentage points (p.p.) of linear spectral unmixing (LSU) without (blue) and with calibration (green) using a superficial vessel, and with fluence compensation (pink) is plotted against the depth of the evaluation pixel for both the whole phantom (top) and for the vessels only (bottom). The bands around the solid lines indicate the standard deviation.

The performance for the three tested oximetry methods (LSU, LSU with calibration from signal correlation analysis, and LSU with prior fluence compensation) applied to both PAT and HSI data can be found in Table 3. On average, the calibrated versions of LSU showed lower MAE and reduced standard deviation compared to the uncalibrated approach, suggesting improved performance. Fluence compensation in PAT increased the accuracy of evaluation in vessels, although its benefit for the entire phantom was similar to the uncalibrated method. A key consideration for PAT is evaluation at a function of tissue depths (Fig. 7) through which our findings indicate that the calibrated LSU outperforms the uncalibrated approach at most depths in the entire phantom; the fluence compensation method particularly performs well for the first 5 mm.

**Table 3:** Mean absolute error for linear spectral unmixing (LSU) applied on photoacoustic tomography (PAT) images and hyperspectral imaging (HSI) images.

	PAT		HSI	
	Entire phantom	Vessels-only	Entire phantom	Vessels-only
LSU	33.4±19.6	30.4±18.4	39.9±15.0	45.3±19.0
	[32.6, 34.1]	[29.4, 31.3]	[39.6, 40.3]	[44.7, 46.0]
Calibrated LSU	29.9±5.0	27.9±4.9	32.0±0.6	31.1±0.8
	[29.7, 30.1]	[27.6, 28.1]	[32.0, 32.0]	[31.1, 31.1]
Fluence compensation	27.9±17.9	24.2±16.9	Not applicable	
	[27.2, 28.6]	[23.3, 25.1]		

Calibrated LSU indicates that the correlation function was calculated via linear regression in Sec. 3.3. For PAT, fluence compensation means that the reconstructed image was corrected based on simulated fluence. The standard deviation is indicated with “±” and the bounds of the 95% confidence intervals of the mean are in square brackets: [low, high]. All values are in percentage points (p.p.).

## 4 Discussion

In this study, we presented a set of anthropomorphic forearm phantoms that were designed to replicate the optical absorption of ${\mathrm{HbO}}_{2}$ and Hb at five distinct ${\mathrm{sO}}_{2}$ levels (0%, 30%, 50%, 70%, and 100%) within a biologically relevant wavelength range (700 to 850 nm). By employing 3D-printed molds and strategically selected dyes, these phantoms achieved a morphologically realistic geometry while reproducing the optical properties ${\mathrm{HbO}}_{2}$ and Hb.

Phantom designs of previous work often rely on simplified geometries such as tubular or multilayer structures. Although flow-based setups for controlled ${\mathrm{sO}}_{2}$ exist, they generally lack morphological accuracy or long-term stability. Our approach addresses these shortcomings by incorporating forearm morphology and carefully chosen dyes. This strategy yields phantoms that more closely match the morphology and absorption characteristics of human tissue and provides a robust testing platform for oximetry methods across various imaging modalities. It also avoids the need for an *in situ* assessment of ground-truth ${\mathrm{sO}}_{2}$, as would be required in a flow circuit. Although there may be instances when testing with blood itself is vital, for example, for optical systems that rely on scattering from moving red blood cells, for many target applications such as intraoperative imaging, optical systems could be more easily and routinely tested using the approach outlined here.

One core contribution of our work is the open-source data and code for both the phantom molds and dye optimization process. Researchers can 3D-print their own molds derived from high-resolution MRI data, ensuring consistent morphology across laboratories. They can also adapt our dye optimization framework to derive new chromophore combinations, extending the utility of our work to other tissue types. Moreover, our validation experiments with HSI and PAT indicate that the fabricated phantoms produce signals in good agreement with measured absorption, thereby offering a reliable, standardized environment not only for validation of different imaging systems between sites but also for evaluating different oximetry algorithms. By providing a standardized but anthropomorphic phantom, the approach described here could accelerate the development and comparison of optical imaging methods, ultimately improving clinical translation.

Our correlation analyses show that both HSI and PAT signals strongly match the known absorption coefficients of each phantom, underscoring the fidelity of the phantoms for experimental validation. In particular, Pearson correlation coefficients consistently exceeded 0.8, and the analysis for the five example forearms demonstrated close alignment with the expected spectral behavior. Some instability was observed when applying oximetry methods at depth, which is likely due to various vessels being embedded at different positions, having substantially larger absorption coefficients than their surroundings. Therefore, when going from background material to vessel material, the error of the oximetry method might experience a sudden drop or increase.

Despite these advantages, several limitations remain. First, the two dyes used exhibit highly nonlinear mixing behavior, complicating the process of achieving intermediate ${\mathrm{sO}}_{2}$ levels. Although we successfully generated five distinct levels, further refinement is needed to enhance reproducibility and minimize iteration. Further, even though we showed that the HS signals did not degrade over time, we did not explicitly test the long-term optical and chemical stability of these dyes within the base copolymer-in-oil matrix. Second, the DIS system employed for optical characterization is susceptible to measurement uncertainties, which have been reported in prior studies.^44^ Although we attempted to minimize these uncertainties by verifying the mixing ratio both by weighing the dyes and by using LSU on the measured $\mu_{a}$, minor deviations are still possible. Because the focus of this paper was on the optical properties of the phantoms, a full acoustic characterization was not performed, and only basic speed of sound measurements, borrowed from previous work,^28^ were used. Finally, the phantoms contained small air bubbles despite extensive vacuuming, which may cause acoustic reverberations, particularly for vessels located deeper than 1.5 cm. Therefore, we recommend limiting the validation of oximetry methods with these phantoms to vessels whose centres lie at depths shallower than 1.5 cm. To simplify fabrication and ensure reproducibility, we opted for a homogeneous background material without an additional skin-mimicking layer. Although adding a realistic skin layer is conceptually valuable and would add more realism to the phantoms, it is technically challenging in a phantom of this size without introducing more air entrapments. Finally, for visual comparison, we showed a side-by-side view of a human forearm, an example forearm phantom, and its corresponding simulation in Fig. S31 in the Supplementary Material, including their unmixed images. As there are still some very apparent differences such as the heterogeneity of the bulk background or the size of vessels, future work will tackle these discrepancies.

## 5 Conclusion

By enabling accurate and repeatable performance assessments, our tissue-mimicking phantoms provide a robust standard for oximetry validation, bridging the gap left by limited *in vivo* reference methods. Looking ahead, the fabrication strategies and dye selection processes described here offer an initial step for developing even more complex phantoms, thereby fostering more reliable and clinically translatable optical imaging techniques.

## Supplementary Material

10.1117/1.JBO.30.7.076006.s01

## Data Availability

The data and code to reproduce the findings of this study are openly available. The data are available under the CC-BY 4.0 license at: https://doi.org/10.5281/zenodo.15102333. The code is available under the MIT license at: https://github.com/IMSY-DKFZ/anthropomorphic-phantoms.
