# Supplementary material for: Anthropomorphic tissue-mimicking phantoms for oximetry validation in multispectral optical imaging
Source: J Biomed Opt. 2025 Jul 17;30(7):076006. doi: 10.1117/1.JBO.30.7.076006 (PMC12267859; doi:10.1117/1.JBO.30.7.076006)
Supplement: Supplementary file 1 [file JBO_030_076006_SD001.pdf]

Anthropomorphic tissue-mimicking phantoms for oximetry validation in multispectral optical imaging: Supplementary material

Kris K. Dreher[a,b,*], Janek Gröhl[c,d], Friso Grace[a,e], Leonardo Ayala[a,f], Jan-Hinrich Nölke[a,g], Christoph J. Bender[a,g], Melissa J. Watt[c,d], Catherine-Louise White[c,d], Ran Tao[c,d], Wibke Johnen[h,i], Minu D. Tizabi[a], Alexander Seitel[a], Lena Maier-Hein[a,f,g,j,†,*], Sarah E. Bohndiek[c,d,†,*]

[a]German Cancer Research Center (DKFZ), Division of Intelligent Medical Systems (IMSY), Heidelberg, Germany
[b]Heidelberg University, Faculty of Physics and Astronomy, Heidelberg, Germany
[c]University of Cambridge, CRUK Cambridge Institute, Cambridge, United Kingdom
[d]University of Cambridge, Department of Physics, Cambridge, United Kingdom
[e]University of St Andrews, School of Physics and Astronomy, St Andrews, United Kingdom
[f]NCT Heidelberg, a partnership between DKFZ and University Hospital Heidelberg, National Center for Tumor Diseases (NCT), Heidelberg, Germany
[g]Heidelberg University, Faculty of Mathematics and Computer Science, Heidelberg, Germany
[h]DKFZ, Division of Medical Physics in Radiation Oncology, Heidelberg, Germany
[i]Heidelberg Institute for Radiation Oncology (HIRO), National Center for Radiation Research in Oncology (NCRO), Heidelberg, Germany
[j]Heidelberg University, Medical Faculty, Heidelberg, Germany

***Corresponding authors: KKD, LMH {k.dreher,l.maier-hein}@dkfz-heidelberg.de, SEB seb53@cam.ac.uk
†Shared last authorship

S1 Supplementary Notes

The phantom manufacturing protocol used for quality assurance during phantom production is embedded below.

Measurement Protocol for Multispectral Photoacoustic Inclusion Materials

Date: ______________________

Operator: ______________________

Experiment ID: ______________________

Colour: ______________________

Phantom preparation protocol

Please check the boxes when a step has been finished!

☐ Step 1: Prepare 30 ml stock solution with 2 mg/ml

Check?	Instruction	Comment	
☐	Weigh 30 ml (25.14 g) of mineral oil in a skirted tube	$M_{measured}$:	g
☐	Weigh 60 mg of the dye powder	$M_{measured}$:	mg
☐	Mix the mineral oil and the dye in the vortexer (30 s)	$T_{vortexed}$:	s
☐	Sonicate in the water bath sonicator for 10 s and then shake the tube by hand. (repeat ~10 times)	$N_{repetitions}$:	
☐	Crush the little bits and clumps of the dye with a spatula.		
☐	Repeat the previous 3 steps until there are no clumps left that are bigger than a granule of sugar.		
☐	Label the tube with the dye ID and the date of production		

☐ Step 2: Prepare phantoms according to multicentre study

The following steps are meant to briefly summarise and amend the IPASC multicenter phantom material verification study 2022 Protocol. This means that the steps are adjusted to a 50 ml volume of mineral oil (conversion ratio of 0.5 from 100 ml to 50 ml). In doubt, please refer to the full study protocol!

Check?	Instruction	Comment	
☐	Prepare a silicone oil bath, set the temperature to 160°C		
☐	Weigh 50 ml (41.90 g) of mineral oil in a beaker (beaker 1)	$M_{measured}$:	g

☐	Vortex the stock solution for 1 min	$T_{vortexed}$:	min
☐	Pipette 12.8 ml of the stock solution	Stock solution:	
☐	Add the stock solution to beaker 1		
☐	Weigh 76.5 mg of TiO2 and add to beaker 1	$M_{measured}$:	mg
☐	Sonicate and sway beaker 1 until no TiO2 is settled at the bottom. This will take roughly 20 - 30 min. In the meantime, the following 3 steps can be performed.	$T_{sonicated}$:	min
☐	Weigh 1.0 g of Butylated Hydroxytoluene	$M_{measured}$:	g
☐	Weigh 12.57 g of SEBS	$M_{measured}$:	g
☐	Add SEBS and Butylated Hydroxytoluene with a magnet stirrer in a new beaker (beaker 2)		
☐	When there is no settled TiO2 left in beaker 1, pour the content of beaker 1 into beaker 2		
☐	Heat beaker 2 in the oil bath for about 45 min with aluminium foil on top	$T_{in\ oil}$:	min
☐	After 10 min, take off the aluminium cover and stir the mixture with a metallic spoon/spatula.		
☐	When the mixture has become liquid, put in vacuum chamber for at least 1 min	$T_{in\ vacuum}$:	min
☐	Scratch residual bubbles from surface and repeat the previous step if necessary		

☐ Step 3: Casting the phantom material into the mold for optical samples

This step is for fabricating the optical samples used to measure absorption and scattering of the material. For this, take four microscopy glass slides, put a metallic frame on two of the slides and pour the material in it. Try to be quick when covering the material and the metallic frame so that the material is still fluid and can be compressed into shape.

S2 Supplementary Figures

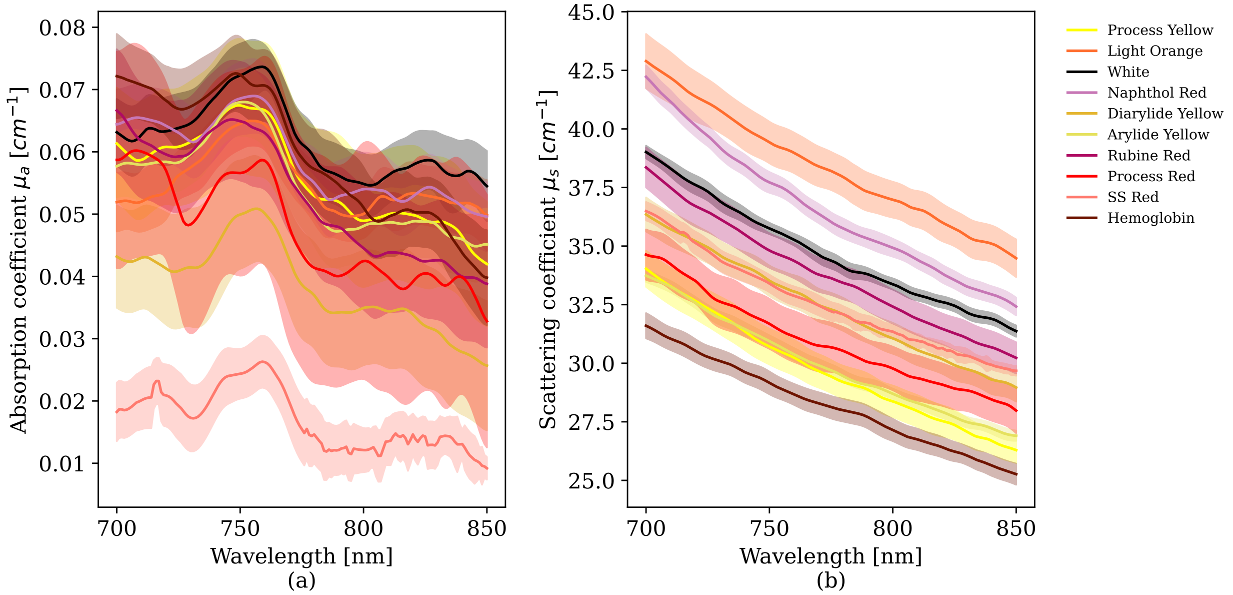

Fig S1: **Optical properties of investigated dyes with low absorption.** In total, 26 dyes were examined for their optical absorption (a) and scattering (b) in a copolymer-in-oil solution using a double-integrating sphere setup. 10 dyes were identified with relatively low absorption coefficient: Process Yellow, Light Orange, White, Naphthol Red, Diarylide Yellow, Rubine Red, Process Red, SS Red, Hemoglobin. For manufacturers and product names, refer to Table S1. Bands around the measured spectra indicate the standard deviation across the 12 measurement points for each optical sample slab.

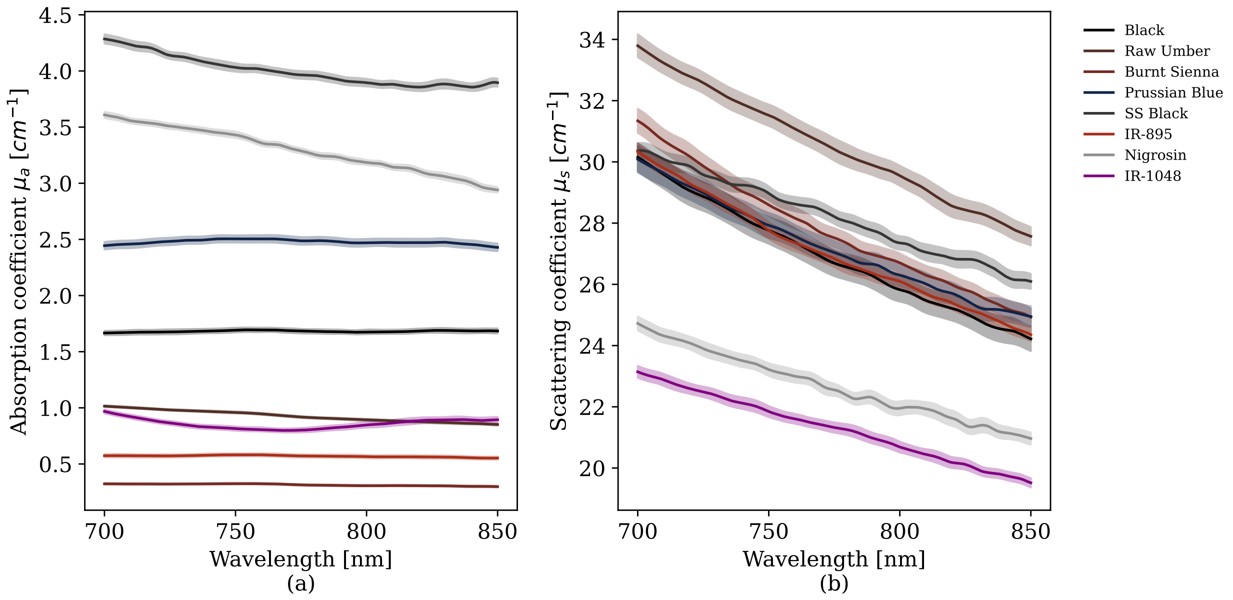

Fig S2: **Optical properties of investigated dyes with high absorption.** In total, 26 dyes were examined for their optical absorption (a) and scattering (b) in a copolymer-in-oil solution using a double-integrating sphere setup. 8 dyes were identified with relatively high absorption coefficient: Black, Raw Umber, Burnt Sienna, Prussian Blue, SS Black, IR-895, Nigrosin, IR-1048. For manufacturers and product names, refer to Table S1. Bands around the measured spectra indicate the standard deviation across the 12 measurement points for each optical sample slab.

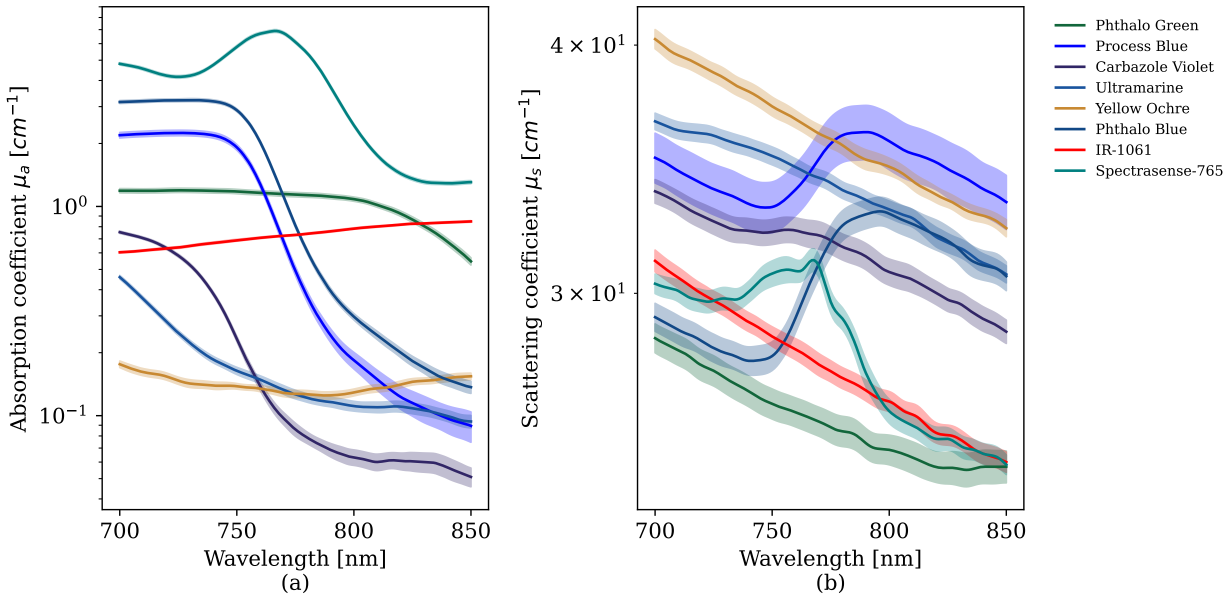

Fig S3: **Optical properties of investigated dyes with high range.** In total, 26 dyes were examined for their optical absorption (a) and scattering (b) in a copolymer-in-oil solution using a double-integrating sphere setup. 8 dyes were identified with a relatively high range of absorption coefficient: Phthalo Green, Process Blue, Carbazole Violet, Ultramarine, Yellow Ochre, Phthalo Blue, IR-1061, Spectrasense-765. For manufacturers and product names, refer to Table S1. Bands around the measured spectra indicate the standard deviation across the 12 measurement points for each optical sample slab.

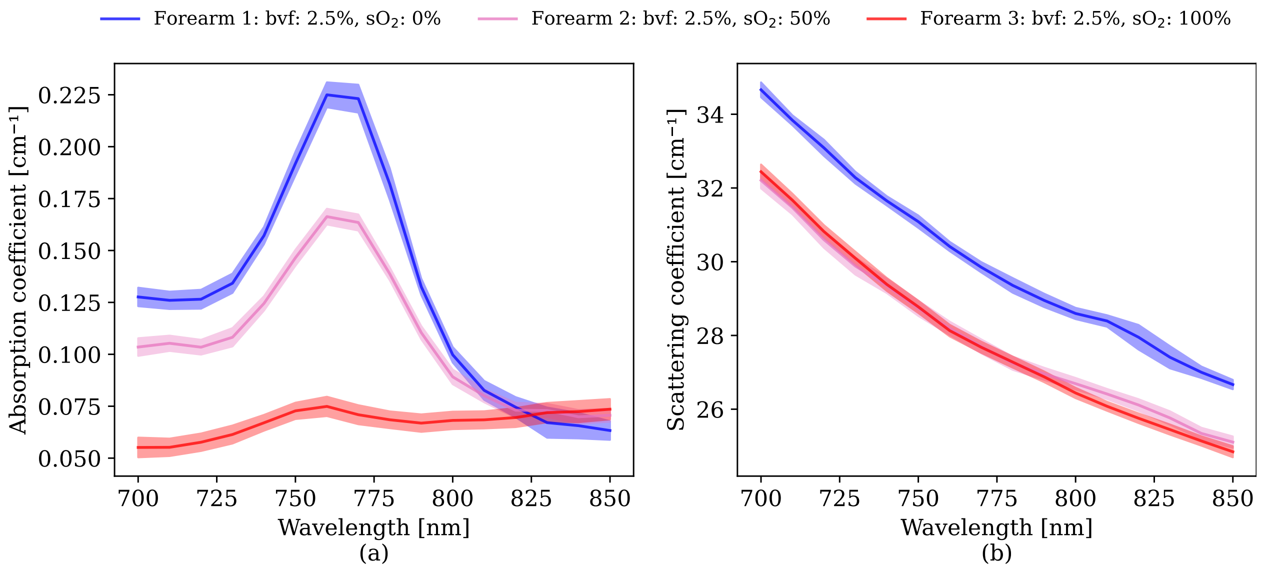

Fig S4: **Optical absorption (a) and scattering (a) coefficients for forearms 1-3.** For scattering anisotropy (g), a value of g=0.7 was assumed.

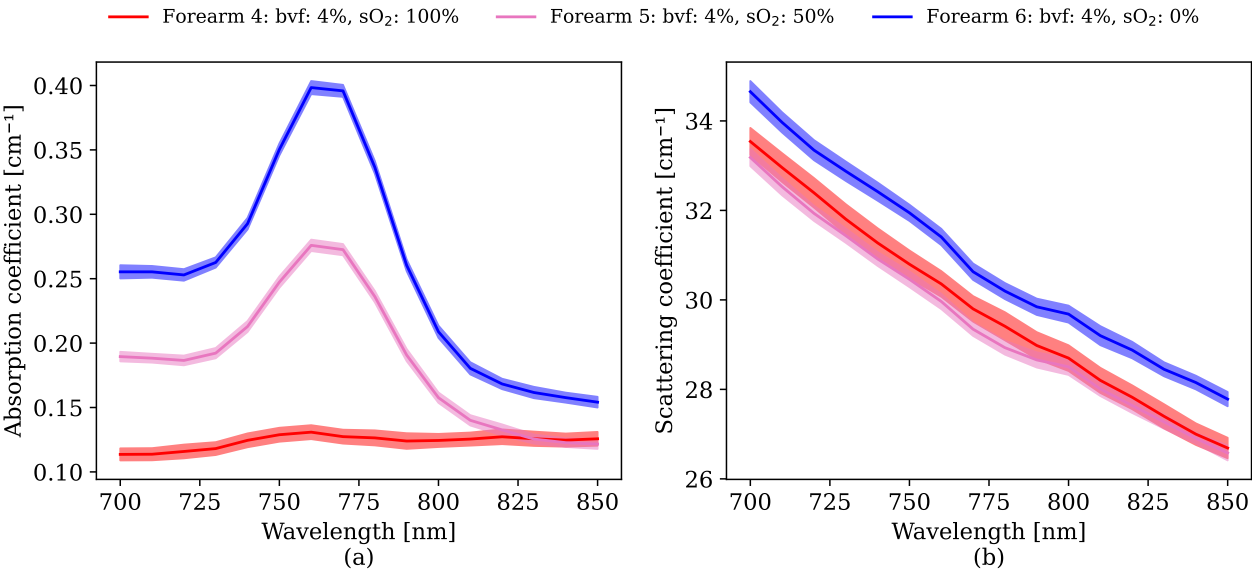

Fig S5: **Optical absorption (a) and scattering (a) coefficients for forearms 4-6.** For scattering anisotropy (g), a value of g=0.7 was assumed.

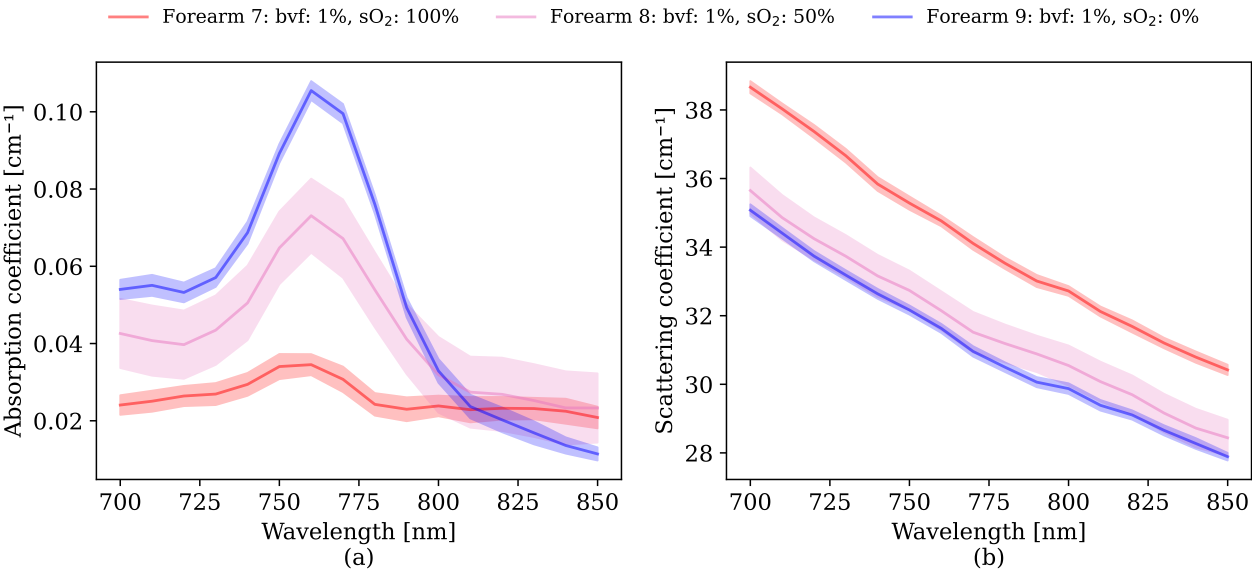

Fig S6: **Optical absorption (a) and scattering (a) coefficients for forearms 7-9.** For scattering anisotropy (g), a value of g=0.7 was assumed.

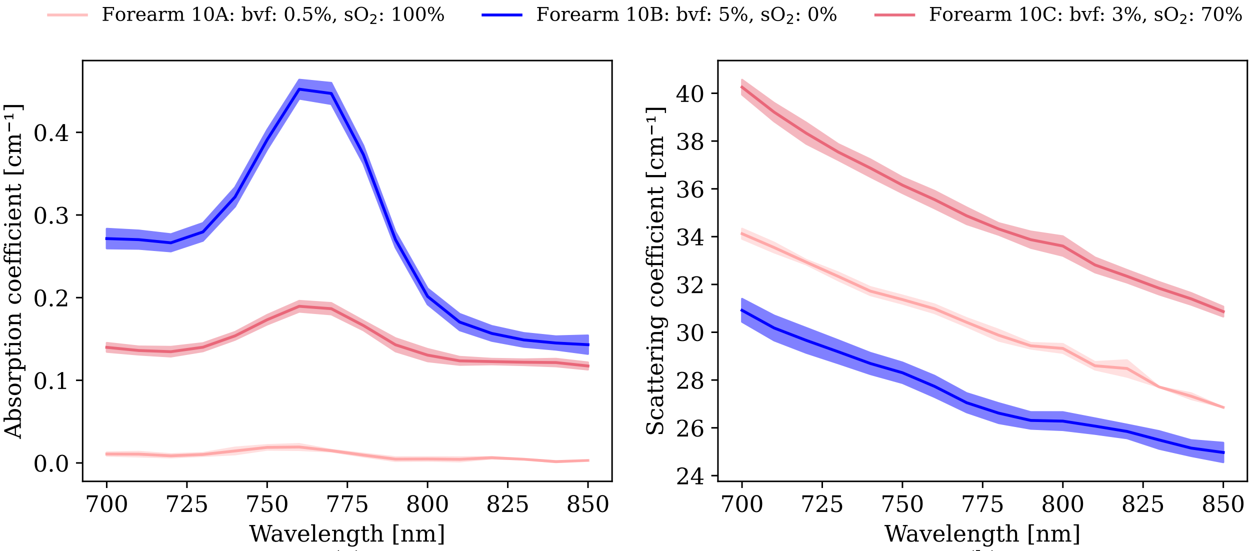

(a) (b)

Fig S7: **Optical absorption (a) and scattering (b) coefficients for all three background compositions of forearm 10 (10A, 10B, and 10C).** For scattering anisotropy (g), a value of g=0.7 was assumed.

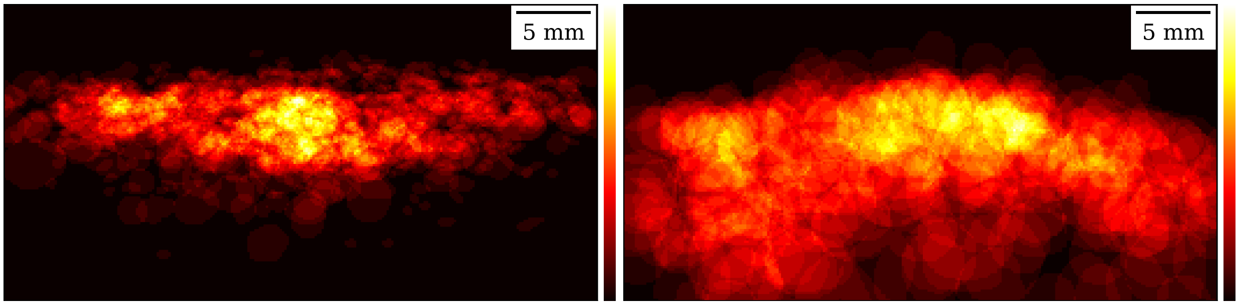

(a) Human forearm vessel locations (b) Phantom forearm vessel locations

Fig S8: **Heatmap comparison of vessel locations in an in-house human forearm dataset and our forearm phantoms.** The vessels in our phantoms cover the broad area that the human forearm vessels cover with similar hotspot locations. They also extend out into depth more to provide more versatility as we expect more vessels in depth but they were not annotated in the human forearms due to low signal.

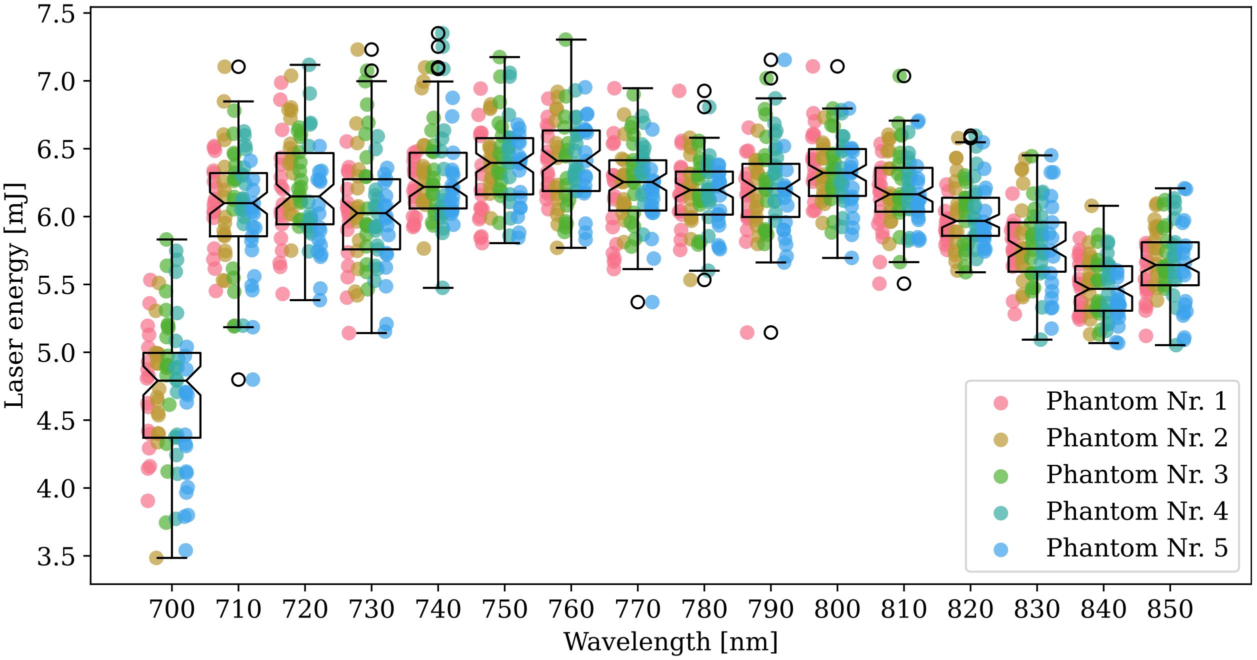

Fig S9: **Measured laser energies in mJ for each of the example phantoms' 27 photoacoustic images.**

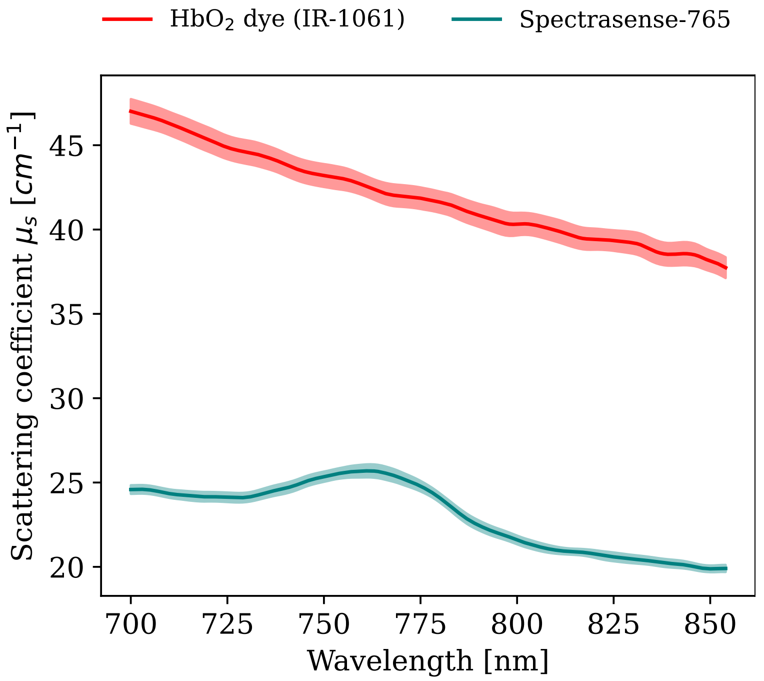

Fig S10: **Scattering spectra of IR-1061 and Spectrasense-765.** Solid lines indicate the measured scattering μ_s spectra of the proxy dyes. Bands around the measured spectra indicate the standard deviation across the 12 measurement points for each optical sample slab.

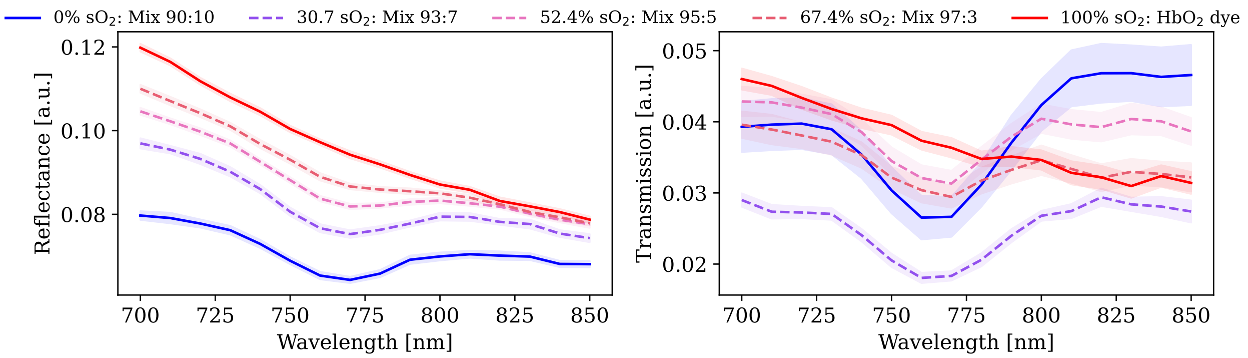

Fig S11: **Raw reflectance and transmission spectra from the DIS system for the five oxygen saturation levels were used for forearm phantom fabrication.** Based on IR-1061 and Spectrasense-765, five oxygen saturation levels (in %) were derived with the respective mixture ratios of 100:0, 97:3, 95:5, 93:7, 90:10. (a) and (b) represent the double integrating sphere measurements of reflectance and transmission, respectively. Solid lines are the spectra that are used as endmembers (0% oxygen saturation and 100% oxygen saturation) for linear spectral unmixing (LSU). Dashed lines represent the intermediate levels and the corresponding percentages in the legend are the LSU results when using the solid lines as endmembers. Bands around the spectra indicate the standard deviation across the 12 measurement points for each optical sample slab.

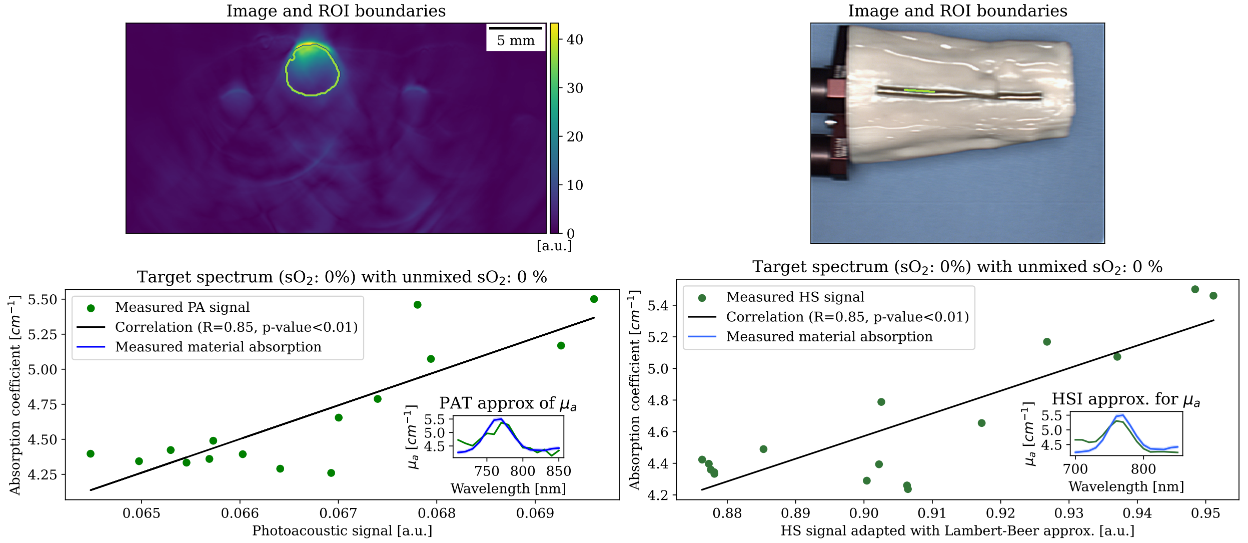

(a) (b)

Fig S12: **Examples of signal correlation of the 0% oxygen saturation superficial vessel for both (a) photoacoustic (PA) tomography (PAT) and (b) hyperspectral (HS) imaging (HSI).** Top pictures show (a) PA image with region of interest (ROI) boundary (green outline) and (b) RGB- (red blue green) rendered HS image with its ROI boundary. Lower pictures show the correlation of measured spectra (green dots as explained in sec. 3 of the main paper) with the measured absorption coefficients. Black solid lines represent the resulting linear regression function with corresponding R values. The inset plots show the measured absorptions (blue) and estimated absorptions (green, using the correlation function) as qualitative confirmation.

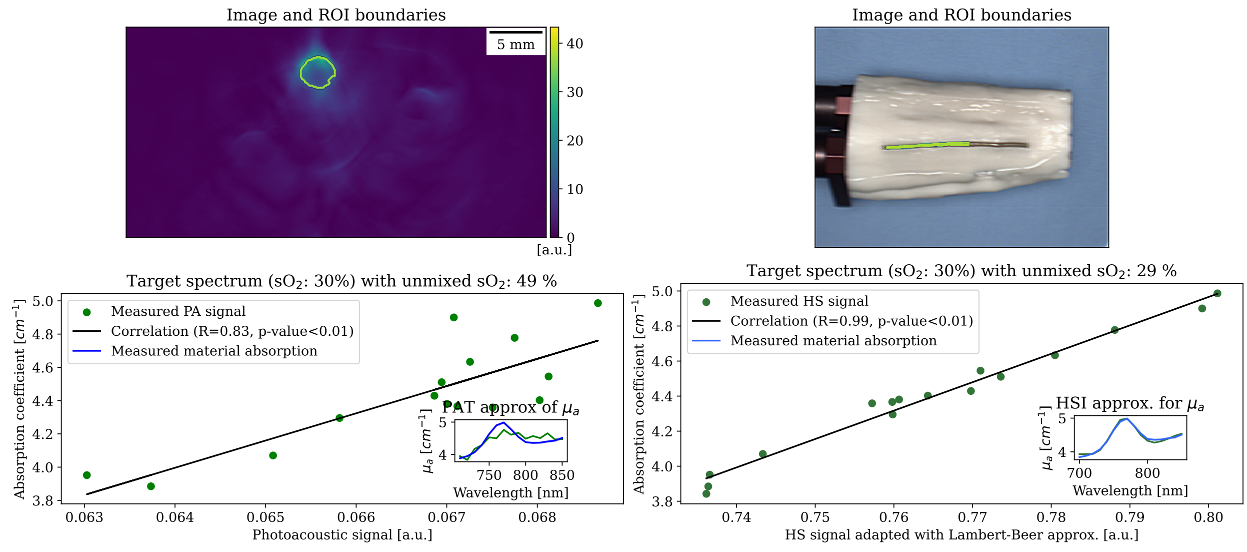

(a) (b)

Fig S13: **Examples of signal correlation of the 30% oxygen saturation superficial vessel for both (a) photoacoustic (PA) tomography (PAT) and (b) hyperspectral (HS) imaging (HSI).** Top pictures show (a) PA image with region of interest (ROI) boundary (green outline) and (b) RGB- (red blue green) rendered HS image with its ROI boundary. Lower pictures show the correlation of measured spectra (green dots as explained in sec. 3 of the main paper) with the measured absorption coefficients. Black solid lines represent the resulting linear regression function with corresponding R values. The inset plots show the measured absorptions (blue) and estimated absorptions (green, using the correlation function) as qualitative confirmation.

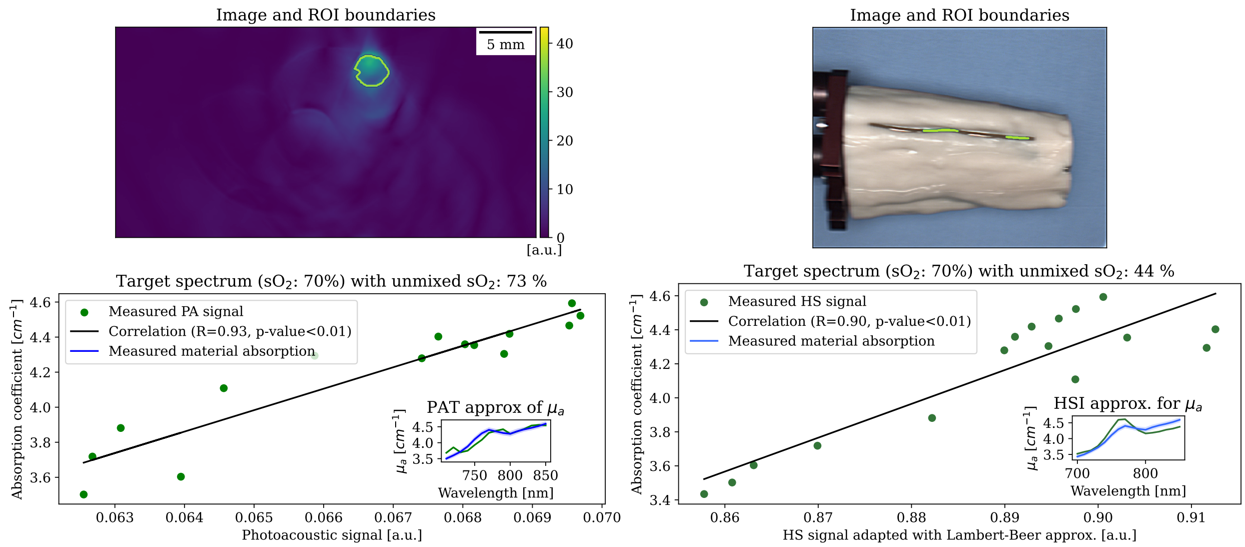

(a) (b)

Fig S14: **Examples of signal correlation of the 70% oxygen saturation superficial vessel for both (a) photoacoustic (PA) tomography (PAT) and (b) hyperspectral (HS) imaging (HSI).** Top pictures show (a) PA image with region of interest (ROI) boundary (green outline) and (b) RGB- (red blue green) rendered HS image with its ROI boundary. Lower pictures show the correlation of measured spectra (green dots as explained in sec. 3 of the main paper) with the measured absorption coefficients. Black solid lines represent the resulting linear regression function with corresponding R values. The inset plots show the measured absorptions (blue) and estimated absorptions (green, using the correlation function) as qualitative confirmation.

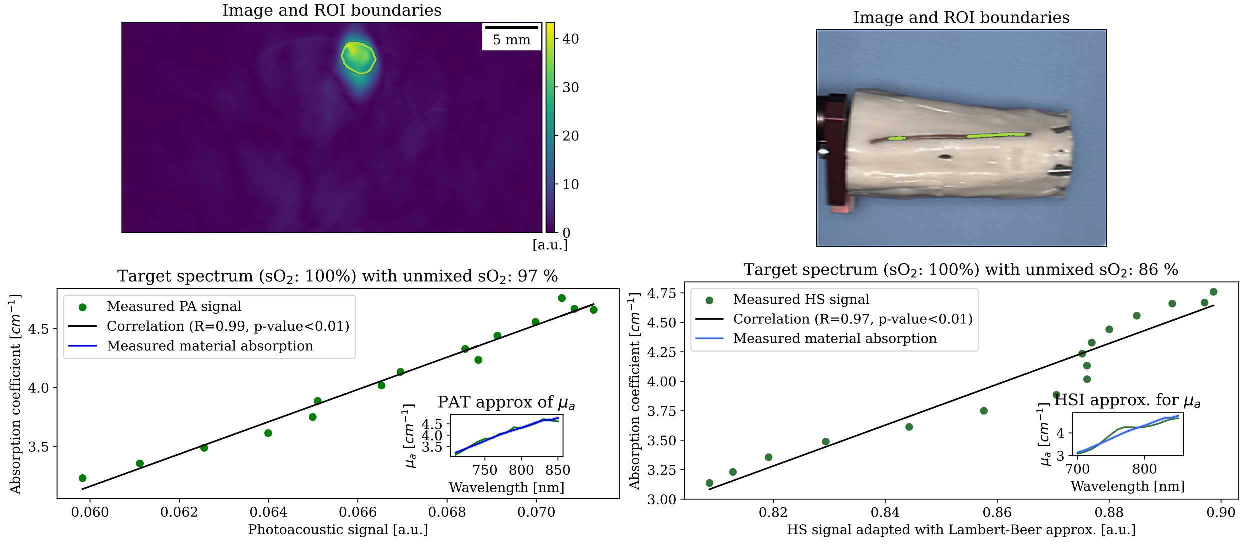

(a) (b)

Fig S15: **Examples of signal correlation of the 100% oxygen saturation superficial vessel for both (a) photoacoustic (PA) tomography (PAT) and (b) hyperspectral (HS) imaging (HSI).** Top pictures show (a) PA image with region of interest (ROI) boundary (green outline) and (b) RGB- (red blue green) rendered HS image with its ROI boundary. Lower pictures show the correlation of measured spectra (green dots as explained in sec. 3 of the main paper) with the measured absorption coefficients. Black solid lines represent the resulting linear regression function with corresponding R values. The inset plots show the measured absorptions (blue) and estimated absorptions (green, using the correlation function) as qualitative confirmation.

S3 Phantom quality assurance

S3.1 Air bubble investigation within phantoms

Our forearm phantoms are considerably larger than many conventional tissue phantoms, making it difficult to achieve uniform fabrication without entrapping air. Indeed, in our experiments, we frequently observed air bubbles within the phantoms. To assess how these inclusions might affect the photoacoustic signal, we performed a small simulation study. First, we segmented example forearms (including visible air bubbles) from ultrasound images. We then simulated photoacoustic images for two conditions, one with and one without the air bubbles, and qualitatively compared the resulting vessel spectra.

Figure S16 illustrates two simulation outcomes for a digital twin of a forearm phantom, depicting scenarios with and without air bubbles. Two vessels, along with their respective oxygen saturation levels, are highlighted, and the mean spectra within each vessel are plotted for both conditions. In superficial vessels, the spectra agreed closely across both models, whereas deeper vessels showed noticeable distortion in the presence of air bubbles. Similar findings were observed in additional forearm phantoms (see Figures S17, S18, S19, and S20). Overall, vessels with centres located deeper than approximately 1.5 cm from the surface appear susceptible to reverberation artefacts caused by the embedded air bubbles.

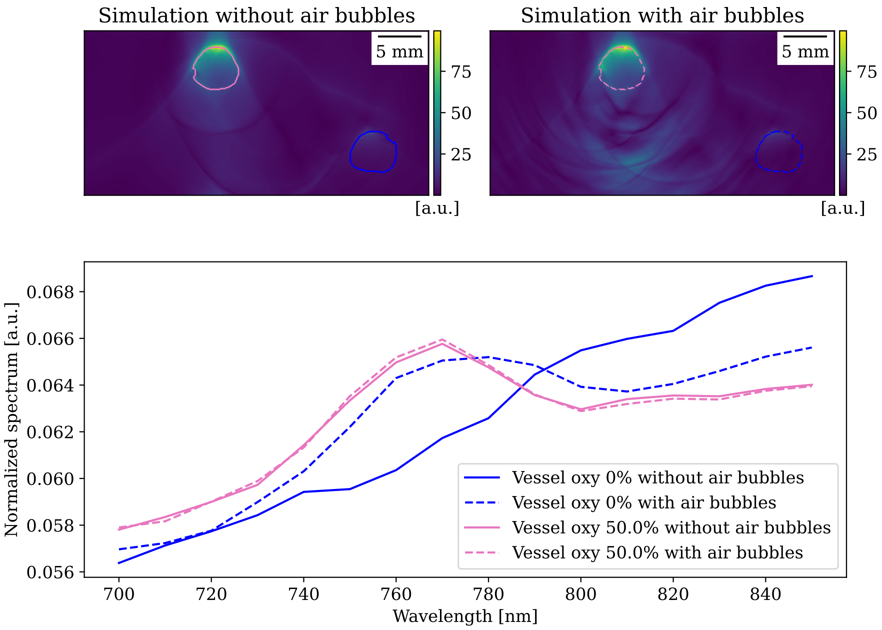

Fig S16: **Image and spectrum comparison of simulations with and without air bubbles.** Photoacoustic images of the forearm phantom 1 were simulated with (top right) and without (top left) air bubbles. Images are shown at 700 nm. Two vessels with different oxygen saturations are marked in the images and their mean spectra are plotted below. The solid lines represent the spectra without air bubbles and the dashed lines represent the spectra with air bubbles.

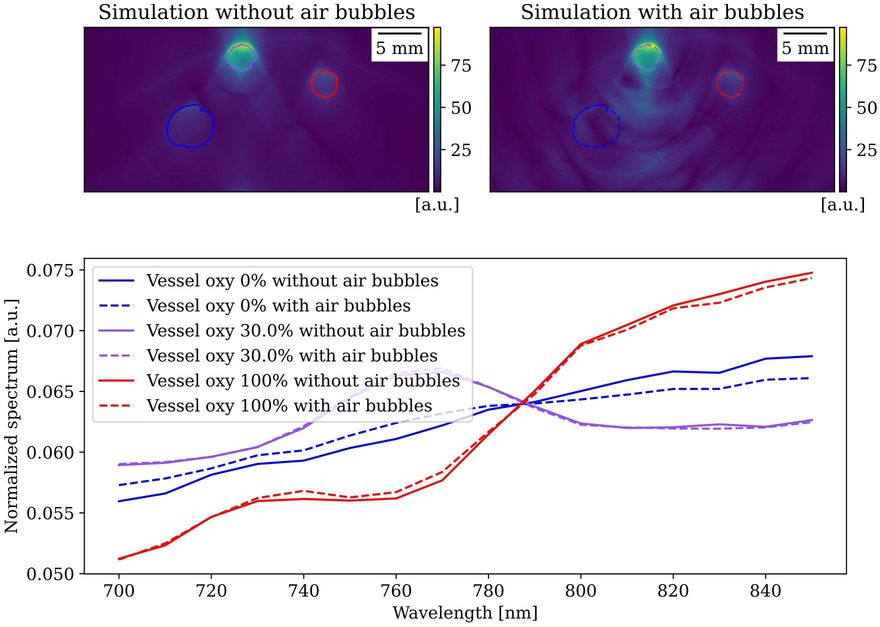

Fig S17: **Image and spectrum comparison of simulations with and without air bubbles.** Photoacoustic images of the forearm phantom 2 were simulated with (top right) and without (top left) air bubbles. Images are shown at 700 nm. Three vessels with different oxygen saturations are marked in the images and their mean spectra are plotted below. The solid lines represent the spectra without air bubbles and the dashed lines represent the spectra with air bubbles.

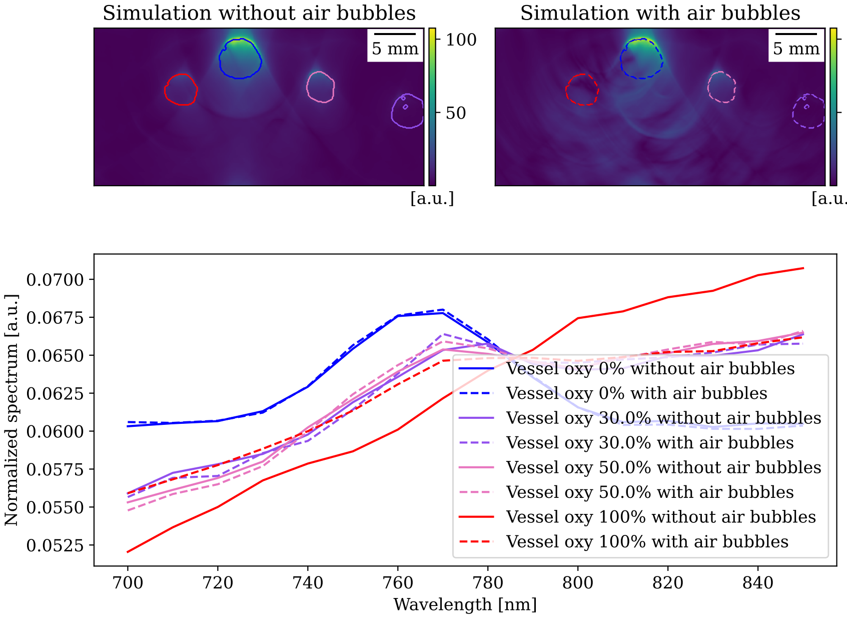

Fig S18: **Image and spectrum comparison of simulations with and without air bubbles.** Photoacoustic images of the forearm phantom 3 were simulated with (top right) and without (top left) air bubbles. Images are shown at 700 nm. Four vessels with different oxygen saturations are marked in the images and their mean spectra are plotted below. The solid lines represent the spectra without air bubbles and the dashed lines represent the spectra with air bubbles. Small purple circles indicate air bubbles.

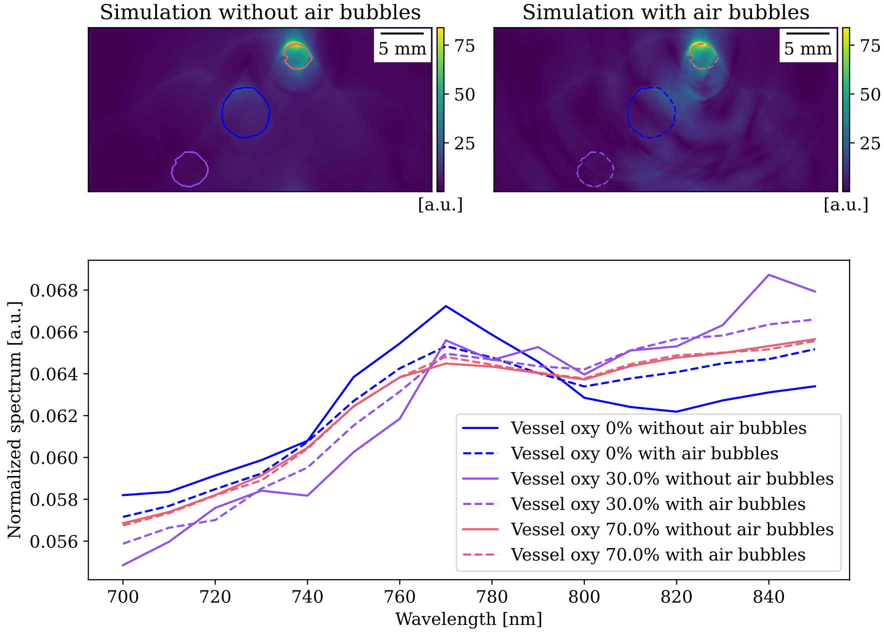

Fig S19: **Image and spectrum comparison of simulations with and without air bubbles.** Photoacoustic images of the forearm phantom 4 were simulated with (top right) and without (top left) air bubbles. Images are shown at 700 nm. Three vessels with different oxygen saturations are marked in the images and their mean spectra are plotted below. The solid lines represent the spectra without air bubbles and the dashed lines represent the spectra with air bubbles.

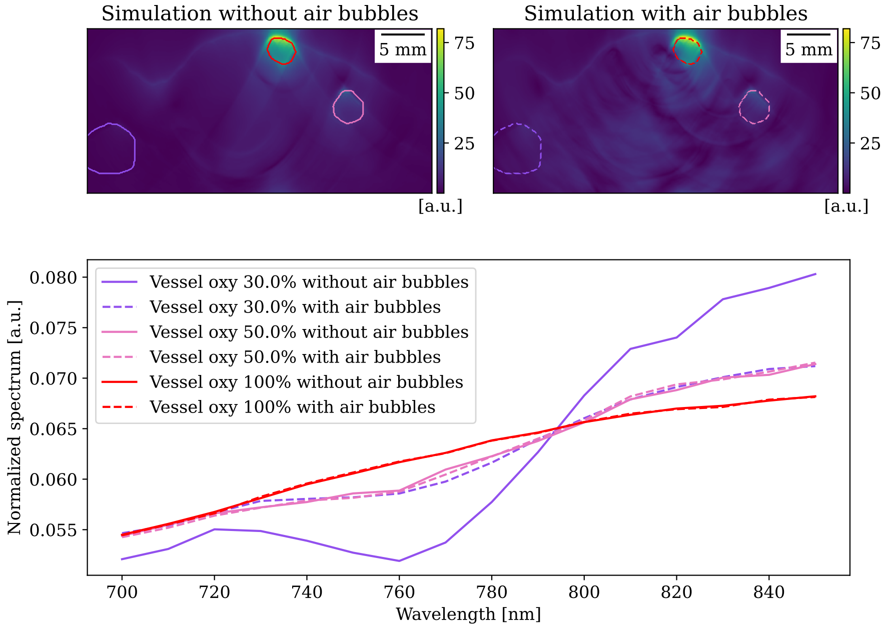

Fig S20: **Image and spectrum comparison of simulations with and without air bubbles.** Photoacoustic images of the forearm phantom 5 were simulated with (top right) and without (top left) air bubbles. Images are shown at 700 nm. Three vessels with different oxygen saturations are marked in the images and their mean spectra are plotted below. The solid lines represent the spectra without air bubbles and the dashed lines represent the spectra with air bubbles.

S3.2 Speed of sound investigation

We assumed a speed of sound of approximately 1470 ms^{-1} in our forearm phantoms. To evaluate whether this approximation is reasonable or significantly off, we conducted a small simulation study using segmentations from the previously described air-bubble investigation. First, we simulated photoacoustic images of the phantoms at 1470 ms^{-1} and then repeated the process with the same segmentations at ±50 ms^{-1} and ±100 ms^{-1} from this baseline value.

Next, we manually segmented superficial vessels in each simulated image according to the previously described protocol, computed the segmented vessel area (A), and derived an approximate vessel diameter (D) from $D = 2\sqrt{A/\pi}$. We compared these diameters to a reference diameter in the image D_{im}, calculated using the fabricated diameter (D_{fab}) and the ratio between the assumed phantom speed of sound (sos_{ref}) and the reconstruction speed of sound (sos_{recon}). Specifically, the reference diameter is given by $D_{\text{im}} = \frac{sos_{\text{recon}}}{sos_{\text{ref}}} D_{\text{fab}}$.

Figure S21 shows the simulation results for a digital twin of a forearm phantom, assuming an sos of 1470 ms^{-1} and variations of ±50 ms^{-1} and ±100 ms^{-1} (for the other phantoms, cf. Figures S22, S23, S24, and S25. In Fig. S21, the fabricated vessel diameter was 5 mm. Under the assumption of 1470 ms^{-1} with a reconstruction at 1497.4 ms^{-1}, we would expect an imaged diameter of 5.09 mm. For the other sos values of -100 ms^{-1}, -50 ms^{-1}, +50 ms^{-1}, and +100 ms^{-1}, the respective imaged diameters were 5.46 mm, 5.27 mm, 4.93 mm, and 4.77 mm. Given the simulated image resolution of 0.1 mm, these findings align closely with theoretical expectations, suggesting that 1470 ms^{-1} is a sufficiently accurate estimate for our phantom material.

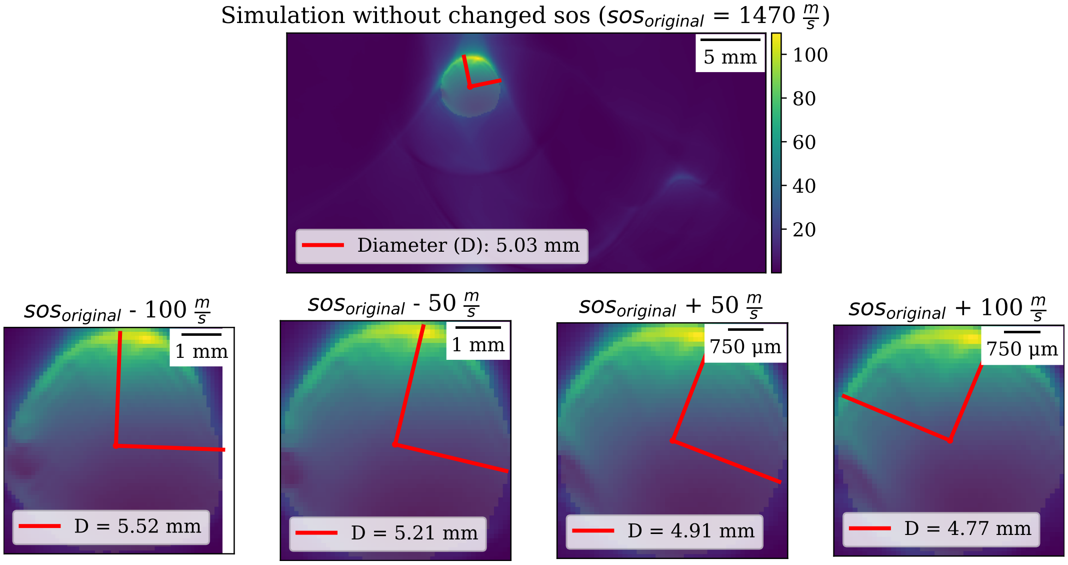

Fig S21: **Vessel diameter comparison for different speeds of sound.** Photoacoustic images of the forearm phantom 1 were simulated with a phantom material speed of sound of 1470 ms^{-1} (top) and then with speeds of sounds that differ from 1470 ms^{-1} by -100 ms^{-1}, -50 ms^{-1}, +50 ms^{-1}, +100 ms^{-1} (from left to right). As the manual segmentations of the superficial vessel were not perfectly circular, the segmentation area A for all images was computed and their diameter D was calculated via $D = 2\sqrt{A/\pi}$. D is indicated in all images by the major axes of the segmented areas.

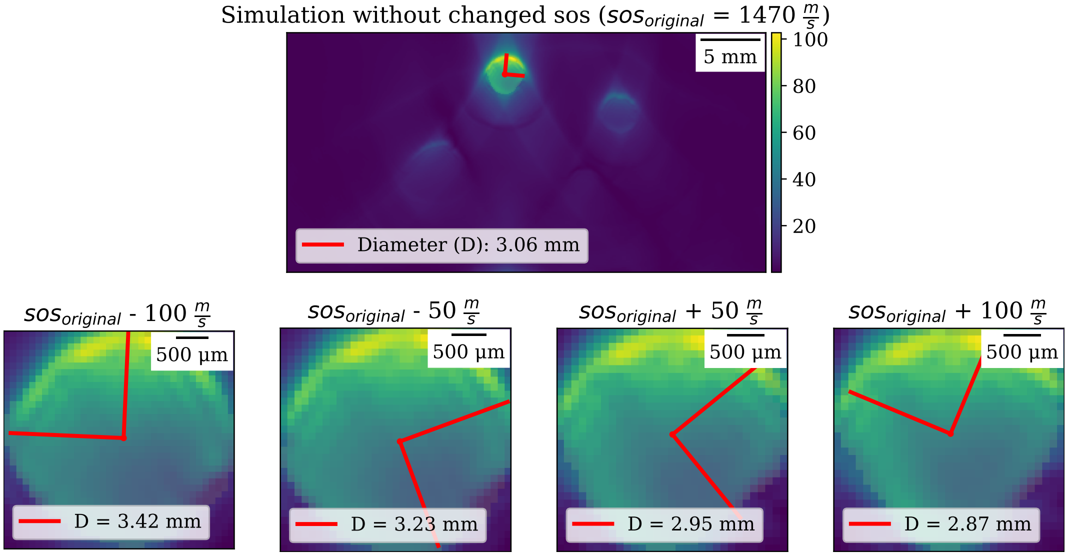

Fig S22: **Vessel diameter comparison for different speeds of sound.** Photoacoustic images of the forearm phantom 2 were simulated with a phantom material speed of sound of 1470 ms^{-1} (top) and then with speeds of sounds that differ from 1470 ms^{-1} by -100 ms^{-1}, -50 ms^{-1}, +50 ms^{-1}, +100 ms^{-1} (from left to right). As the manual segmentations of the superficial vessel were not perfectly circular, the segmentation area A for all images was computed and their diameter D was calculated via $D = 2\sqrt{A/\pi}$. D is indicated in all images by the major axes of the segmented areas.

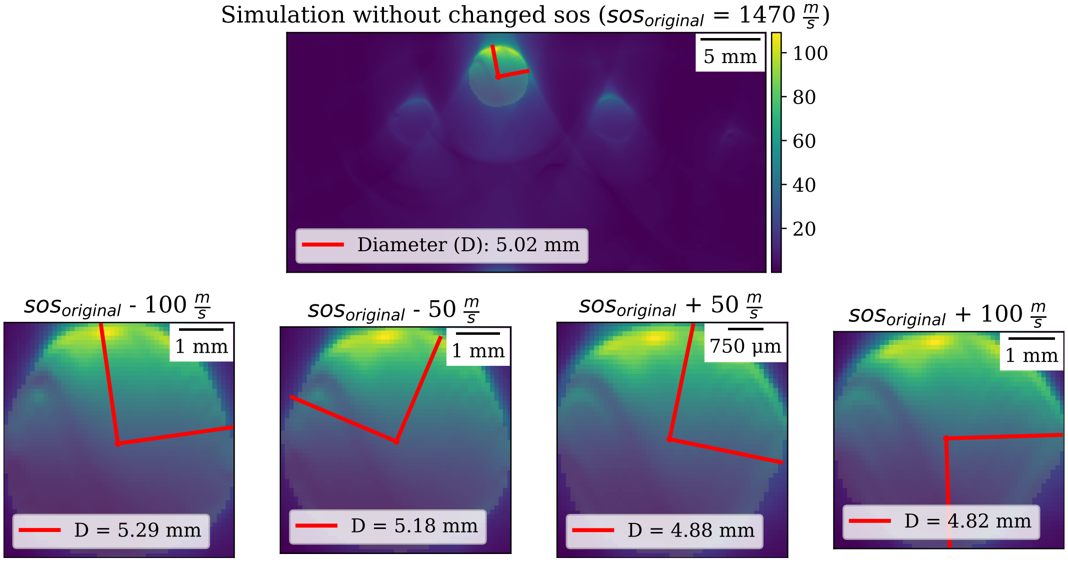

Fig S23: **Vessel diameter comparison for different speeds of sound.** Photoacoustic images of the forearm phantom 3 were simulated with a phantom material speed of sound of 1470 $\mathrm{ms^{-1}}$ (top) and then with speeds of sounds that differ from 1470 $\mathrm{ms^{-1}}$ by -100 $\mathrm{ms^{-1}}$, -50 $\mathrm{ms^{-1}}$, +50 $\mathrm{ms^{-1}}$, +100 $\mathrm{ms^{-1}}$ (from left to right). As the manual segmentations of the superficial vessel were not perfectly circular, the segmentation area A for all images was computed and their diameter D was calculated via $D = 2\sqrt{A/\pi}$. D is indicated in all images by the major axes of the segmented areas.

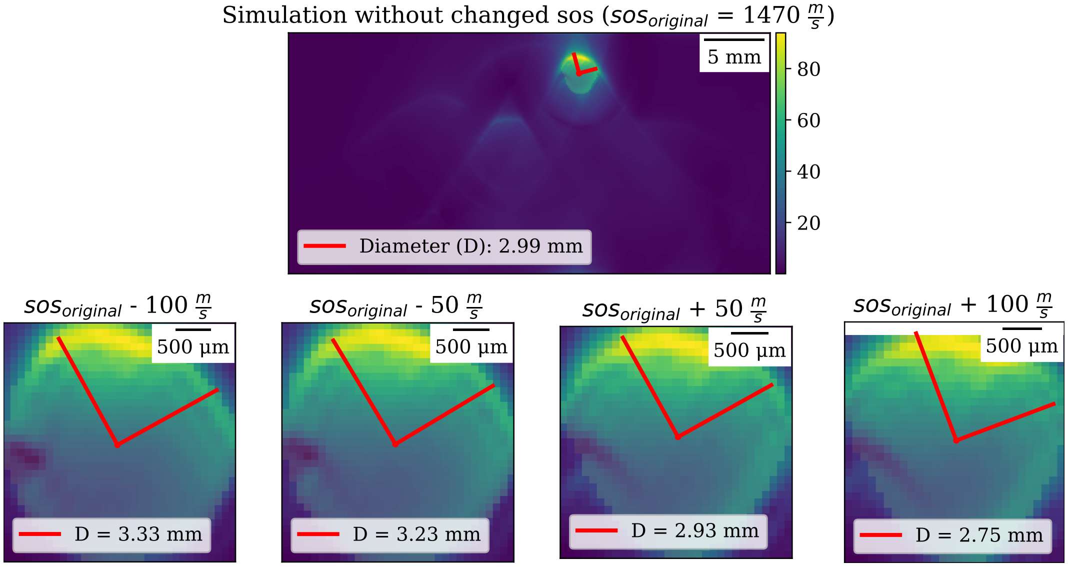

Fig S24: **Vessel diameter comparison for different speeds of sound.** Photoacoustic images of the forearm phantom 4 were simulated with a phantom material speed of sound of 1470 $\mathrm{ms^{-1}}$ (top) and then with speeds of sounds that differ from 1470 $\mathrm{ms^{-1}}$ by -100 $\mathrm{ms^{-1}}$, -50 $\mathrm{ms^{-1}}$, +50 $\mathrm{ms^{-1}}$, +100 $\mathrm{ms^{-1}}$ (from left to right). As the manual segmentations of the superficial vessel were not perfectly circular, the segmentation area A for all images was computed and their diameter D was calculated via $D = 2\sqrt{A/\pi}$. D is indicated in all images by the major axes of the segmented areas.

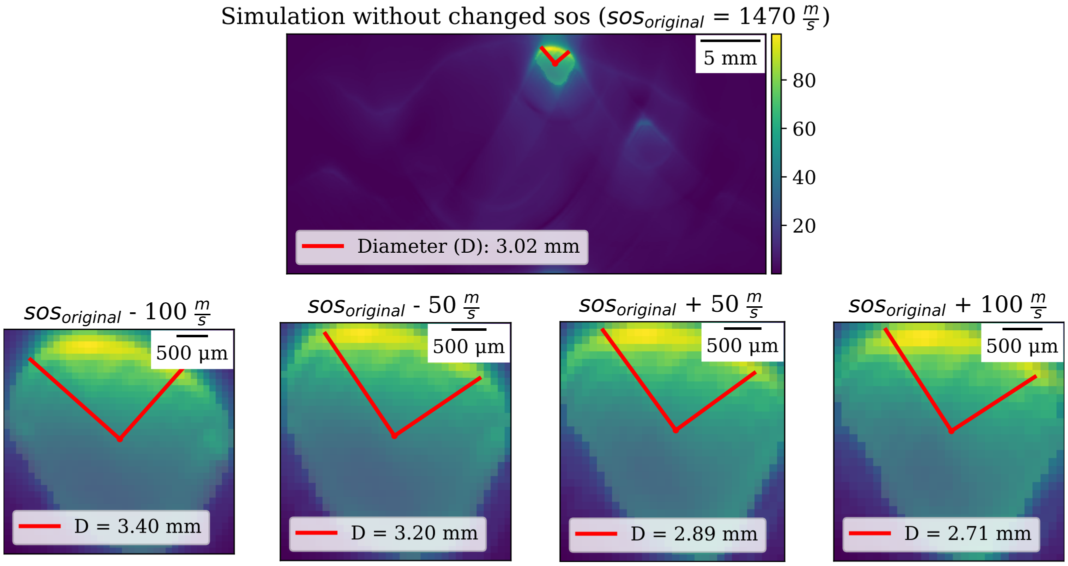

Fig S25: **Vessel diameter comparison for different speeds of sound.** Photoacoustic images of the forearm phantom 5 were simulated with a phantom material speed of sound of 1470 ms^{-1} (top) and then with speeds of sounds that differ from 1470 ms^{-1} by -100 ms^{-1}, -50 ms^{-1}, +50 ms^{-1}, +100 ms^{-1} (from left to right). As the manual segmentations of the superficial vessel were not perfectly circular, the segmentation area A for all images was computed and their diameter D was calculated via $D = 2\sqrt{A/\pi}$. D is indicated in all images by the major axes of the segmented areas.

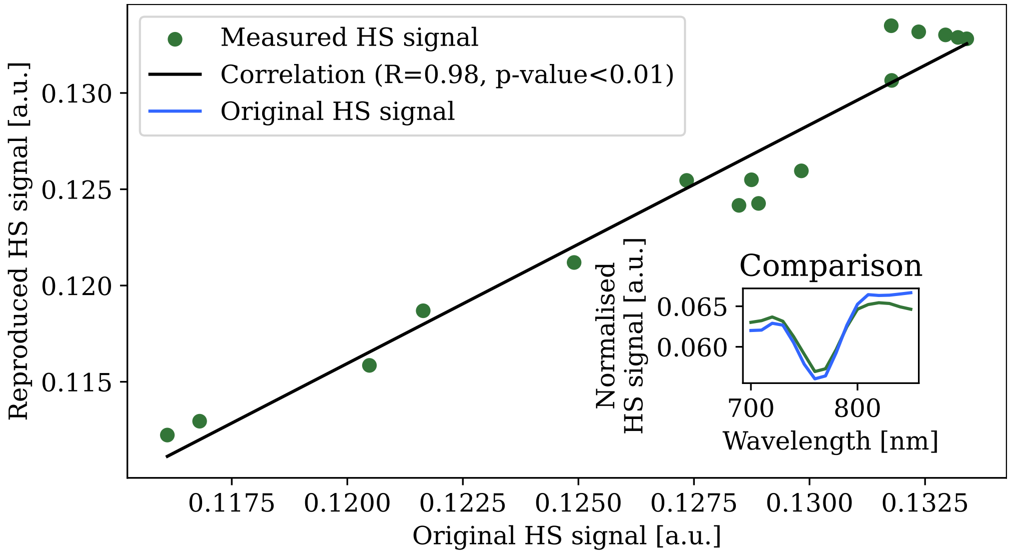

Fig S26: **Signal correlation between the original measured hyperspectral (HS) signal and the re-recorded signal of the 0% oxygen saturation superficial vessel.** Green dots represent the reproduced HS signal and the black solid line represents the resulting linear regression function with corresponding R value. The inset plot shows the normalised re-recorded signal and original signal (blue) as qualitative confirmation.

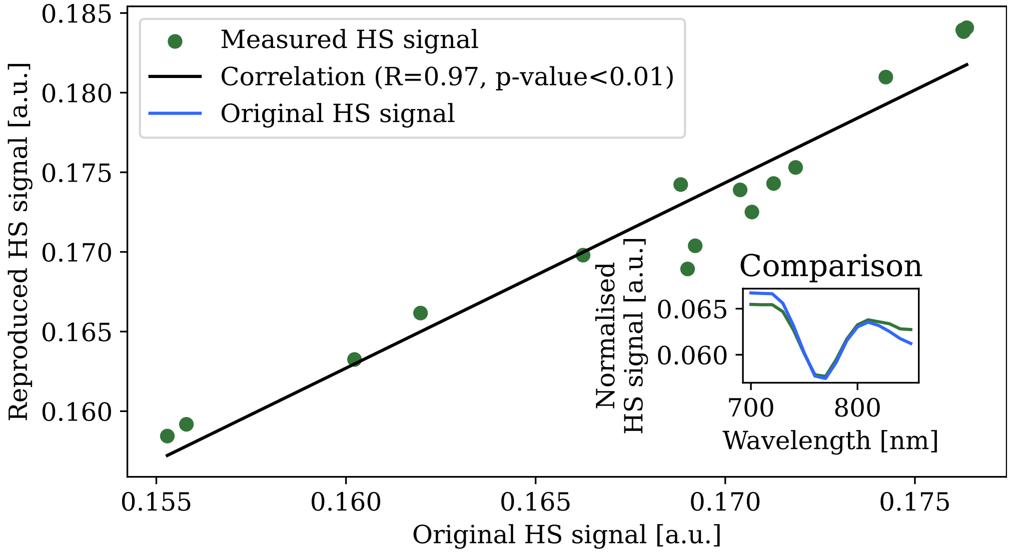

Fig S27: **Signal correlation between the original measured hyperspectral (HS) signal and the re-recorded signal of the 30% oxygen saturation superficial vessel.** Green dots represent the reproduced HS signal and the black solid line represents the resulting linear regression function with corresponding R value. The inset plot shows the normalised re-recorded signal and original signal (blue) as qualitative confirmation.

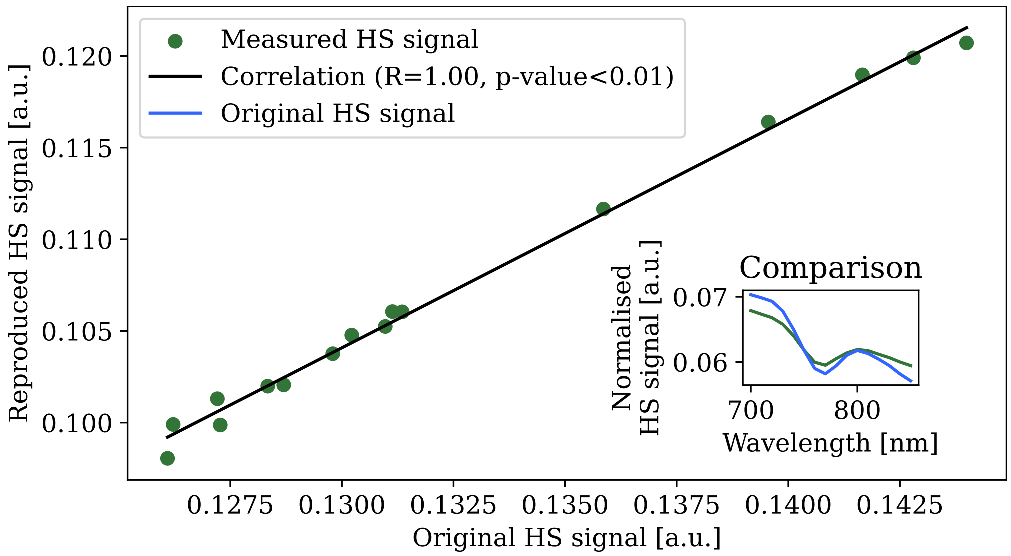

Fig S28: **Signal correlation between the original measured hyperspectral (HS) signal and the re-recorded signal of the 50% oxygen saturation superficial vessel.** Green dots represent the reproduced HS signal and the black solid line represents the resulting linear regression function with corresponding R value. The inset plot shows the normalised re-recorded signal and original signal (blue) as qualitative confirmation.

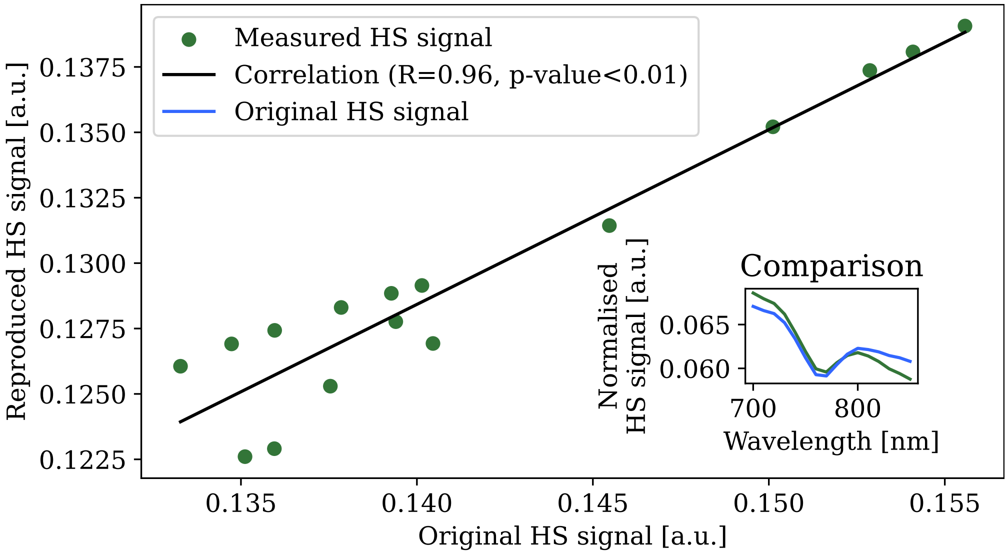

Fig S29: **Signal correlation between the original measured hyperspectral (HS) signal and the re-recorded signal of the 70% oxygen saturation superficial vessel.** Green dots represent the reproduced HS signal and the black solid line represents the resulting linear regression function with corresponding R value. The inset plot shows the normalised re-recorded signal and original signal (blue) as qualitative confirmation.

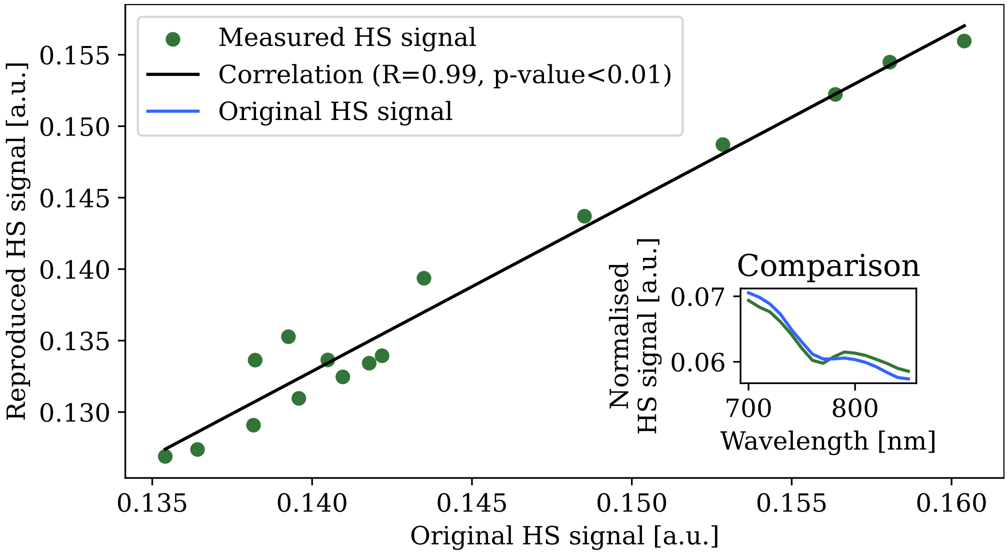

Fig S30: **Signal correlation between the original measured hyperspectral (HS) signal and the re-recorded signal of the 100% oxygen saturation superficial vessel.** Green dots represent the reproduced HS signal and the black solid line represents the resulting linear regression function with corresponding R value. The inset plot shows the normalised re-recorded signal and original signal (blue) as qualitative confirmation.

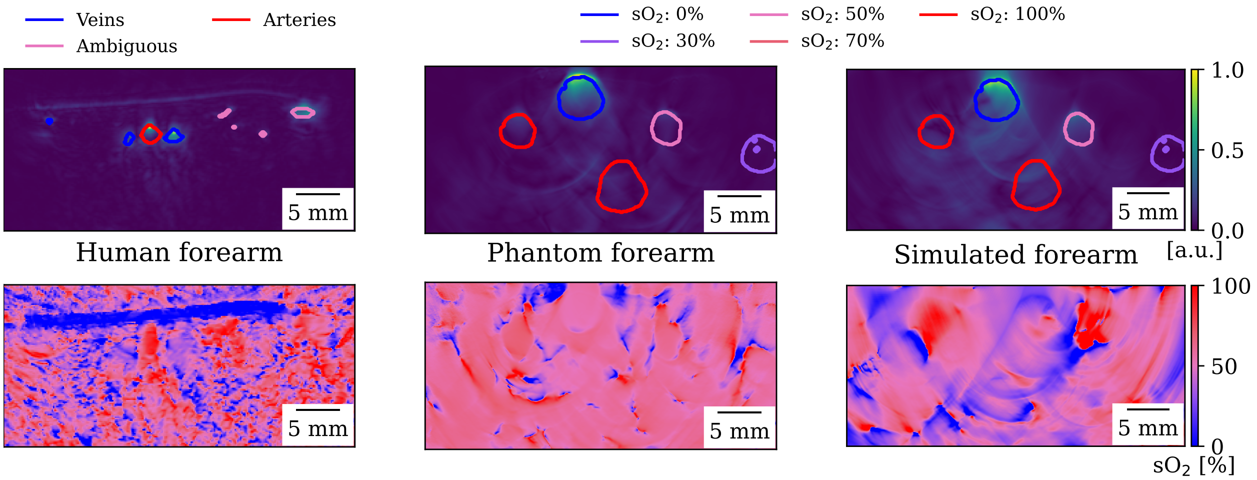

Fig S31: **Side-by-side comparison of a human forearm, an example forearm phantom and its corresponding simulation.** In the upper row, photoacoustic (PA) images with their segmented vessels are presented and in the lower row, the corresponding unmixed images. As we do not have a ground truth for the annotations in the human forearm image, we divided the type of vessels in arteries, veins and ambiguous vessels where we were uncertain. For phantom and simulated forearm, we could annotate with ground truth. We used deoxyhaemoglobin (Hb) and oxyhaemoglobin (HbO_2) as endmembers for the human forearm and our dye proxies as endmembers for the phantom and simulated image.

S4 Supplementary Tables

Dye name	Manufacturer/Vendor	Product name
Black	Cranfield (Jackson's art)	RCR2501860
Process Blue	Cranfield (Jackson's art)	RCR25025291
Process Red	Cranfield (Jackson's art)	RCR25063827
Process Yellow	Cranfield (Jackson's art)	RCR25091884
Arylide Yellow	Cranfield (Jackson's art)	RCR25091630
Diarylide Yellow	Cranfield (Jackson's art)	RCR25091759
Light Orange	Cranfield (Jackson's art)	RCR25091637
Rubine Red	Cranfield (Jackson's art)	RCR25063254
Naphthol Red	Cranfield (Jackson's art)	RCR50063266
Carbazole Violet	Cranfield (Jackson's art)	RCR50071139
Ultramarine	Cranfield (Jackson's art)	RCR50024283
Prussian Blue	Cranfield (Jackson's art)	RCR25024309
Phthalo Blue	Cranfield (Jackson's art)	RCR25024760
Phthalo Green	Cranfield (Jackson's art)	RCR25043104
Yellow Ochre	Cranfield (Jackson's art)	RCR25091737
Burnt Sienna	Cranfield (Jackson's art)	RCR50032371
Raw Umber	Cranfield (Jackson's art)	RCR25032211
White	Cranfield (Jackson's art)	RCR25083391
SS Black	Culture Hustle (Stuart Semple)	BLACK 1.0 Pigment- 50g
SS Red	Culture Hustle (Stuart Semple)	THE WORLD'S PINKEST PINK - 50g
Hemoglobin	Sigma-Aldrich (Merck)	H2625-100G
Nigrosin	Sigma-Aldrich (Merck)	211680-100G
IR-895	Sigma-Aldrich (Merck)	392375
IR-1048	Sigma-Aldrich (Merck)	405175-500MG
IR-1061	Sigma-Aldrich (Merck)	405124-250MG
Spectrasense-765	Sun Chemical Colors & Effects GmbH	Spectrasense™ IR 765

Table S1: Dyes that were investigated for their optical absorption and scattering spectra. The column ”Dye name” indicates the name which is used in this work to refer to the specific dye and the ”Product name” indicates the name that is used by the manufacturer/vendor.

	1	2	3	4	5	6	7	8	9	10A	10B	10C
sO_2	0%	50%	100%	100%	50%	0%	100%	50%	0%	100%	0%	70%
Bvf	2.5%	2.5%	2.5%	4%	4%	4%	1%	1%	1%	0.5%	5%	3%
sO_2 vessel	50%	30%	0%	70%	100%	100%	/	/	/	/	/	/

Table S2: Oxygen saturation (sO_2) and blood volume fraction (Bvf) levels for the background material of each forearm phantom. Bvf refers to the percentage of the total volume that is made up of the dye mixture. While all the others have as uniform as possible properties, the 10th forearm was separated into three parts, with different properties, named 10A, 10B, and 10C. Six of the forearms have a superficial vessel, the oxygen saturation level of which is also reported.

	Hb dye	HbO_2 dye	30% sO_2	50% sO_2	70% sO_2
R-value	1.00	0.90	1.00	1.00	1.00

Table S3: Pearson correlation coefficients (R-values) of the absorption spectra of proxy dyes and consequent oxygen saturation (sO_2) levels with the target absorption coefficients. The proxy dyes were directly correlated with the absorption spectra of Hb and HbO_2. The sO_2 levels 30%, 50%, and 70% were correlated with their target spectrum generated by $\mu_a^{\text{target}} = \text{sO}_2 * \mu_a^{\text{HbO}_2\, dye} + (1 - sO_2) * \mu_a^{\text{Hb dye}}$.
